# HPVE6-USP46 Mediated Cdt2 Stabilization Reduces Set8 Mediated H4K20-Methylation to Induce Gene Expression Changes

**DOI:** 10.3390/cancers14010030

**Published:** 2021-12-22

**Authors:** Shashi Kiran, Briana Wilson, Shekhar Saha, Julia Ann Graff, Anindya Dutta

**Affiliations:** 1 Department of Biochemistry and Molecular Genetics, University of Virginia, Charlottesville, VA 22908, USA; bw9bj@virginia.edu (B.W.); ss7st@virginia.edu (S.S.); jag7dr@virginia.edu (J.A.G.); 2 Department of Biochemistry, School of Life Sciences, University of Hyderabad, Hyderabad 500046, India; 3 Department of Genetics, University of Alabama, Birmingham, AL 35294, USA

**Keywords:** USP46, HPV, E6, deubiquitinase, cervical cancer

## Abstract

**Simple Summary:**

Cervical cancer is one of the most common cancers in women. More than 95% of the cervical cancers are caused by the human papilloma viruses. Earlier we had identified a protein target, USP46 that can be inhibited to block the cancer growth. We found that the viral E6 and human USP46 complex increases the levels of Cdt2 in cervical cancers. In this paper we compared real cervical tumors with matched normal tissue from same patients and found that the effects of E6 mediated USP46 activation are indeed reflected as increased levels of Cdt2 and decreased levels of Set8 protein in the real tumors. We also found that the HPV protein E6 alone can stimulate the enzymatic activity of human USP46. By doing this it activates several cellular pathways that are required for the growth of cancers. One such pathway is the EGFR pathway that is normally upregulated in cervical cancers. We find that E6-USP46 contributes to the activation of EGFR by inducing epigenetic changes on the DNA by degrading the Set8 protein. These findings illuminate the role of viral E6 protein in inducing cancers and substantiate the candidature of USP46 as a drug target for HPV induced cancers.

**Abstract:**

E6 from high-risk strains of HPV is well known to transform cells by deregulating p53. We reported that in HPV transformed cell-lines E6 from high-risk HPV can recruit the USP46 deubiquitinase to substrates such as Cdt2 and stabilize the latter, and that USP46 is important for growth of HPV induced tumors in xenografts. Here we show that in cervical cancer biopsies the stabilization of Cdt2 in the HPV-induced cancers leads to the decrease of a CRL4-Cdt2 substrate, the histone H4K20 mono-methyltransferase Set8, and decrease in H4K20me1 or H4K20me3 that can be detected by immunohistochemistry. In HPV-transformed cancer cell lines *in vitro*, knockdown of E6 decreases Cdt2 and increases Set8. Co-knockdown of Set8 shows that some of the gene expression changes produced by E6 knockdown is due to the increase of Set8. EGFR and EGFR regulated genes were identified in this set of genes. Turning to the mechanism by which E6 stabilizes Cdt2, we find that a purified E6:USP46 complex has significantly more de-ubiquitinase activity in vitro than USP46 alone, demonstrating that E6 can directly interact with USP46 in the absence of other proteins and that it can substitute for the known activators of USP46, UAF1 and WDR20. Deletion mapping of Cdt2 shows that there are three discrete, but redundant, parts of the substrate that are essential for stabilization by E6: USP46. The helix–loop–helix region or the WD40 repeat driven beta-propeller structure of Cdt2 are dispensable for the stabilization implying that interaction with DDB1 (and the rest of the CRL4 complex) or with the substrate of the CRL4-Cdt2 E3 ligase is not necessary for E6:USP46 to interact with and stabilize Cdt2. The identification of 50 amino acid stretches in the 731 amino acid Cdt2 protein as being important for the stabilization by E6 underlines the specificity of the process. In summary, E6 activates the deubiquitinase activity of USP46, stabilizes Cdt2 utilizing multiple sites on Cdt2, and leads to degradation of Set8 and changes in gene-expression in HPV-transformed cells.

## 1. Introduction

Human papilloma viruses (HPV) are responsible for 5% of all cancers in the world and 95% of cervical cancers [1,2,3]. HPV-E6 and E7 are the two viral proteins responsible for its oncogenicity. Whereas E7 de-regulates cell cycle by binding to and inactivating pRb, E6 binds to E6AP, an E3 ubiquitin ligase to degrade p53 [4,5,6]. Three key features demonstrate their importance in HPV induced cancers. (i) HPV-E6 and E7 are sufficient to induce oncogenic transformation [7,8,9,10]. (ii) Sustained expression of E6 and E7 is maintained in HPV induced cancers [11]. (iii) Depletion of E6 or E7 leads to permanent growth arrest [12,13,14]. Overall, HPV induced cancers are addicted to HPV E6 and E7 oncoprotein, and hence these proteins are specific targets for therapy of such cancers.

We reported that HPV-E6 recruits USP46, a human deubiquitinase (DUB) to form an E6-USP46 complex, which targets proteins for stabilization by deubiquitination [15]. USP46 belongs to ubiquitin specific proteases of DUB family and is involved in replication and pathologies of cancer-causing viruses [15,16,17]. USP46 is enzymatically inactive by itself and requires cofactors like UAF1 and WDR20 for its activity, but co-immunoprecipitation experiments suggested that the E6:USP46 complex does not contain UAF1 or WDR20 [15,18,19]. DUBs are important targets for therapy because they have enzymatic pockets that can be targeted by small molecule drug inhibitors to inhibit their catalytic activity. HPV-E6 exists as multimers and binds to E6AP to degrade several cellular substrates [20,21]. We identified Cdt2, an oncogenic protein to be the target of the E6-USP46 complex. Cdt2 is a substrate adaptor of the CRL4-Cdt2 E3 ubiquitin ligase. It interacts with the CRL4 complex through DDB1 and recruits many targets of CRL4 like the cell cycle regulators p21, Set8 and Cdt1 through physical interaction with the targets. Set8 is a methyltransferase and the only known mono-methyltransferase for histone H4K20. H4K20 methylations and Set8 levels are regulated by the cell cycle [22]. The monomethylated H4K20 (H4K20-me1) is methylated further by other methytransferases to produce the H4K20 tri-methyl mark (H4K20-me3), which is seen near epigenetically repressed genes. Loss of H4K20-me3 is often seen in cancers [23].

In the present work we answer four outstanding questions. First, are the changes in Set8 and in H4K20me1 or H4K20me3 that are predicted to occur from our experiments in HPV-transformed cell-lines, actually seen in cervical cancer biopsies? Second, does the degradation of Set8 lead to measurable changes in gene expression in HPV transformed cells? Third, does pure recombinant E6 activate recombinant USP46 in the complete absence of known cellular activators of USP46 like UAF1 and WDR20? Finally, are there specific parts of the Cdt2 protein that are critical for the stabilization by E6: USP46, and if so, do they indicate that Cdt2 has to be associated with CRL4 or with substrates of CRL4 for Cdt2 to be stabilized by E6:USP46? The answers support the presence of the proposed E6-USP46-Cdt2-Set8-H4K20me1 signal transduction process in carcinogenesis by HPV and set the stage for screening for inhibitors of USP46 that will be useful for therapy of such cancers.

## 2. Results

### 2.1. E6-USP46 Mediated Stabilization of Cdt2 Leads to Set8 Degradation and Loss of H4K20 Methylation in HPV Positive Cancers

**Figure 1:**
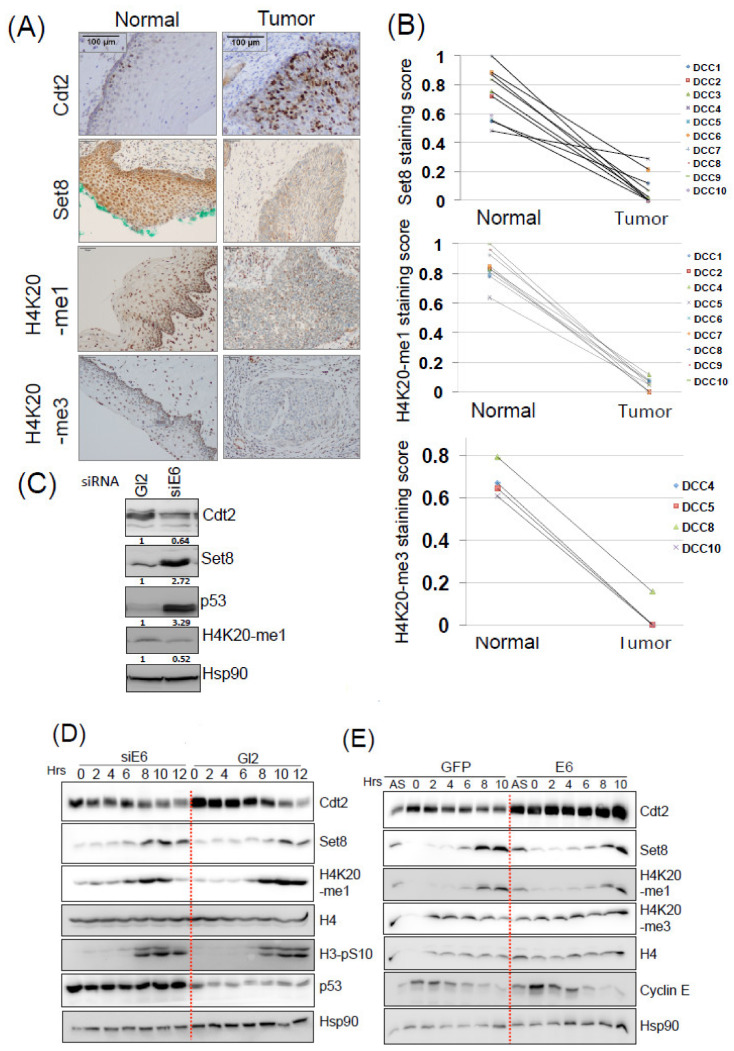
(**A**) Immunohistochemical staining of Cdt2, Set8, H4K20-me1 and H4K20-me3 cells in cervical cancer and adjacent normal tissues. (**B**) Summary of staining scores for Set8, H4K20-me1 and H4K20-me3 in normal and cancerous tissue. DCC 1–10 denotes individual patients for each normal and cancer pairs. (**C**) HeLa cells were transfected with control (Gl2) or indicated siRNAs and collected 48 h after transfection. The cell lysates were then immunoblotted with indicated antibodies. (**D**) HeLa cells were transfected twice with control (Gl2) or HPV-E6 siRNAs before and during the double thymidine block (DTB). Cells were collected at indicated time points after release from DTB and subjected to immunoblotting with indicated antibodies. (**E**) HeLa cells were transfected with GFP or HPV-16E6 expressing plasmids followed by double thymidine block (DTB). Cells were collected at indicated time points after DTB and subjected to immunoblotting with indicated antibodies.

We have already reported that Cdt2 protein is increased in cervical cancers [15]. To determine if the stabilization of Cdt2 by E6-USP46 alters Cdt2 substrates in cervical cancers, we checked the expression of Set8 in cancer biopsies. Ten samples of matched cancerous and adjacent normal tissues were analyzed by immunohistochemistry for Set8. The specificity of the anti-Set8 antibody was ascertained by absence of staining in a Set8 knockout cell line (Supplementary Figure S1A). The expression levels of Set8 were greatly reduced in HPV-positive cancers compared to adjacent normal tissue. This was seen in all 10 samples (Figure 1A,B). Note that Ki-67 staining showed the expected increase in cell proliferation in the cancers relative to normal epithelium (Supplementary Figure S1B). To determine whether the decrease of Set8 affects H4K20-me1 or H4K20-me3 levels, these sections were stained with H4K20-me1 and H4K20-me3 antibodies by immunohistochemistry. The levels of H4K20 –me1 and -me3 were also greatly reduced in these cancers compared to the adjacent normal tissue (Figure 1A,B). Although we could not stain for E6 due to technical reasons, since 95% of cervical cancers are caused by HPV, the reproducible changes in multiple cervical cancers together with the experimental results reported by us in [15], supports the hypothesis that the changes reported here are due to the HPV E6 present in cervical cancers.

We turned to HPV positive cell line, HeLa, to check if the observation in these cancers can be modeled in vitro. We knocked down HPV-E6 and looked for changes in Set8 and H4K20-me1 levels. Depletion of E6 for 48 hr led to significant up-regulation of p53, indicating the efficiency of the E6 knockdown. Cdt2 was decreased and Set8 increased, but there was no significant increase in H4K20 mono-methylation in the cell-cycle asynchronous cell population during the period of the experiment (Figure 1C).

Set8 and H4K20 methylation levels are upregulated in late S and G2 phases of the cell-cycle [22]. To check if cell cycle stage specific changes in the expression of Set8 and H4K20-me1 are induced by transient depletion of E6, we synchronized HeLa cells (normally expressing E6) at the G1-S transition by double thymidine block during which time they were treated with the siGL2 (negative control) or siE6. The depletion of E6 was confirmed be demonstrating the resulting induction of p53 protein, which is normally degraded by E6. The cells were then chased for 12 h after release from the block (Figure 1D). Phosphorylation of histone H3 in the later time points (G2 and M) shows that the cells were synchronized in their progression through S phase into M. Set8 was increased in late S and G2 in the control cells (10–12 h after release) treated with siGL2 but there was a 2–4 h earlier increase in Set8 in S phase upon E6 knockdown. There was a very mild increase in H4K20-me1 during the earlier parts of S phase, that were most evident at 2–6 h after release (Figure 1D).

Conversely, E6 protein was transiently overexpressed in similarly cell-cycle synchronized HeLa, and here the induction of Cdt2, stabilized by E6 serves as a surrogate marker for the E6 overexpression. Vectors overexpressing GFP were used on the negative control cells. In this case the cell-cycle synchrony during and after release from double thymidine block was confirmed by the transient expression of cyclin E only during the early time-points (early S phase) (Figure 1E). E6 overexpression led to stabilization of Cdt2 but only a slight decrease of Set8 at the time-points in late S when Set8 is normally at its maximum. There was a very slight decrease in the H4K20-me1 levels in the last two time-points, near the G2/M transition, but since there was no decrease of H4K20-me3 level at those time-points, the me1 level changes were most likely insignificant. Note the apparent decrease of H4K20 methylation at time 0 in the control (GFP overexpressing) cells is an artifact due to under loading of H4 in that lane.

These results indicate that while a change in Cdt2 levels is seen in 24–48 h after altering HPV-E6 expression, the downstream effects on Set8 and H4K20 methylation require passage of the cells through many cell-cycles when E6 is over-expressed or depleted. Thus, the decrease in H4K20-me1 and -me3 in HPV positive tumors is a result of long-term expression of HPV-E6 expression in HPV positive tumors. Note that no other mono-methyltransferase for H4K20 methylation is known.

### 2.2. E6-USP46 Mediated Set8 Downregulation Is Required for Induction of EGFR and a Subset of Its Target Genes

**Figure 2:**
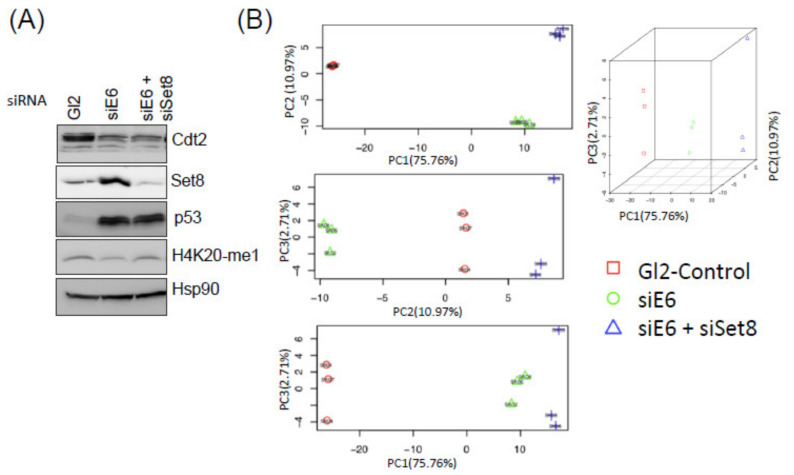

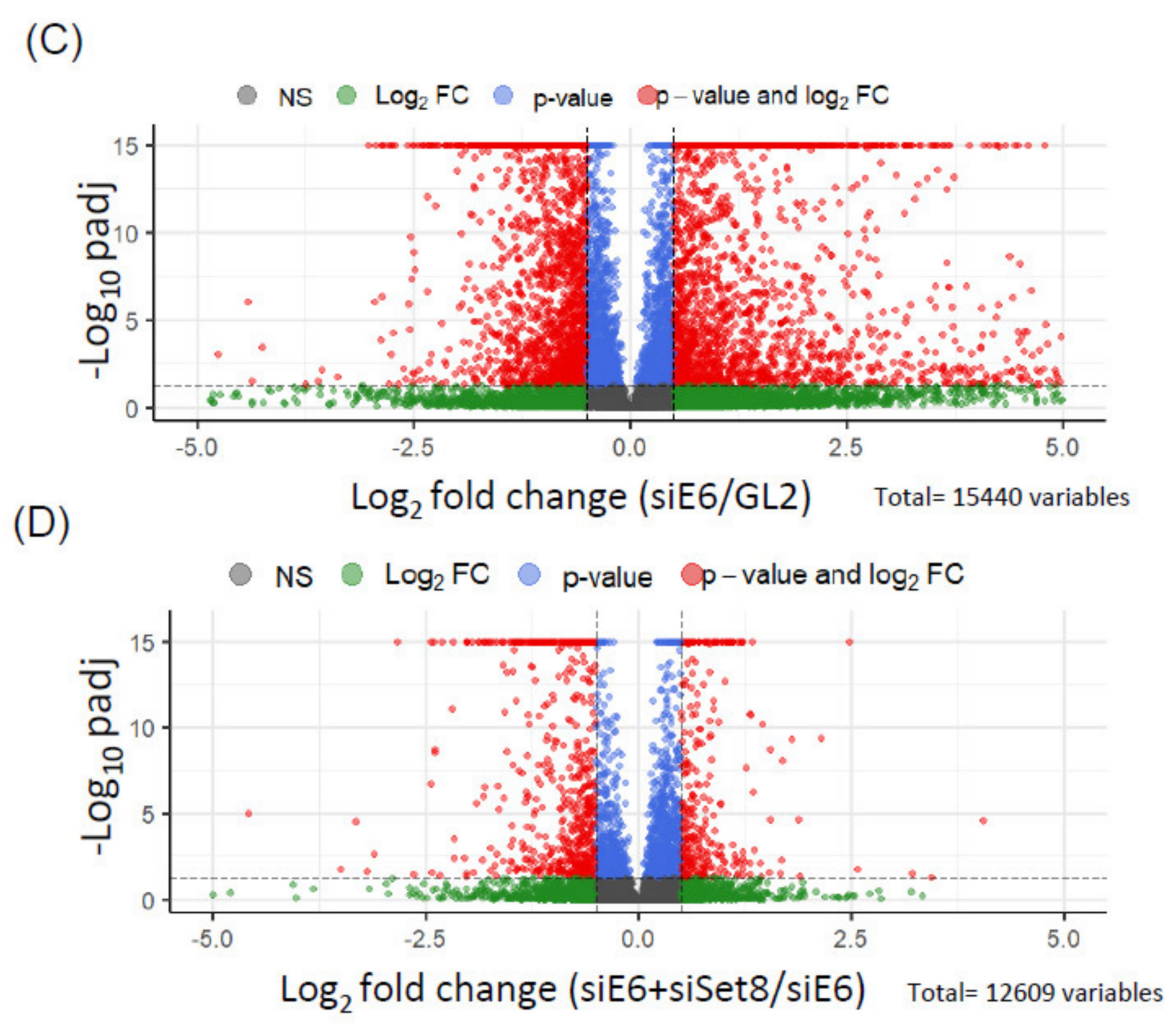
(**A**) Immunoblotting of indicated proteins after siE6 or siE6+siSet8. (**B**) Principal component analysis of the RNA seq results which show that the RNA seq from the knockdown conditions are reproducible and cluster as expected. (**C**,**D**) Volcano plots with Log2 fold change (*X*-axis) vs. −Log10 *p*-value (*Y*-axis) show that many genes are significantly up or down-regulated when E6 is knocked down (**C**) or when Set8 is co-knocked down with E6, relative to E6 alone (**D**). Red dots: genes with Log2FC > |0.5| and −Log10 adjusted *p*-value> 1.3. Genes with a −Log10 *p*-value > 15 were plotted at 15.

The evident decrease of Set8, H4K20-me1 & H4K20-me3 in cervical cancers (Figure 1A,B) suggests an important role of E6-USP46 mediated Set8 degradation in oncogenic progression. The HPV E6 and E7 oncogenes are produced from the same transcript. We knocked down either E6/E7 alone, which would stabilize Set8, or E6/E7 and Set8 together in HeLa cells to identify pathways that are controlled by Set8 and thus are deregulated by E6 through the decrease of Set8. Cell lysates were prepared from a fraction of these cells and checked for Cdt2 depletion and p53 induction indicating the efficacy of the E6 knockdown in both these situations. We also saw induction of Set8 following E6 depletion and Set8 decrease following co-depletion of both E6 and Set8 (Figure 2A). Again, the changes in H4K20me1 levels were subtle during the short term of this experiment leading us to expect only very mild changes in gene expression through this pathway. RNAseq yielded ~70 million reads with 95% mapping rate from each of the samples (Supplementary Tables S1 and S2). Principal Component Analysis shows that the samples produced reproducible results that were well separated by knockdown condition (Figure 2B), indicating high quality RNA sequencing. In the PC2 dimension only, accounting for ~11% of the variability, siE6 moved the RNA-seq in one direction relative to siGL2, but the co-knockdown of Set8 reversed that movement, suggesting that the Set8 increase after E6 depletion is responsible for a small but detectable amount of gene expression change even during these transient changes. siE6 caused a significant alteration in the expression of many genes (log2 FC < −0.5 or >0.5 and −log10 adjusted *p*-value > 1.3) (Figure 2C) as did Set8 knockdown in E6 depleted cells (Figure 2D).

E6 degrades p53, and so as expected, the p53 Hallmark pathway was significantly up-regulated upon E6/E7 depletion and this was still seen after the co-knockdown of Set8, suggesting that Set8 has no role in the downregulation of p53 (Supplementary Figure S2A,B). Similarly, E7 inactivates Rb and turns on E2F, and so the E2F target Hallmark pathway was down-regulated by depletion of E7 (E6/7) (Supplementary Figure S2A). Although this downregulation of E2F targets persisted when Set8 was co-depleted, the decrease was less and did not reach statistical significance. (Supplemental Figure S2A). This suggests that Set8 depletion in E6/E7 transformed cells may have a secondary role in the upregulation of E2F targets, even though the inactivation of Rb by E7 is the primary cause for the upregulation.

Next, we looked for pathways whose dysregulation after siE6/E7 was reversed after co-depletion of E6/E7 and Set8. Of the ten pathways significantly upregulated by siE6/E7, eight were still significantly upregulated after co-depletion of Set8. The pathways involved in myogenesis and apical junction were the only pathways upregulated by siE6/E7 that change direction in being downregulated (though not significantly) upon co-depletion of Set8 (Supplementary Figure S2A,B). Thus, it is possible that the degradation of Set8 by E6, contributes to the repression of myogenesis and apical junction pathways in HPV transformed cells. When we looked at individual genes on these pathways, however, there was only one gene, NEGR1 from the hallmark apical junction pathway that had significant activation after siE6/E7. Here, we are defining significance as an adjusted *p*-value < 0.05 and a log2 fold change in gene expression in both conditions greater than +/− 0.5. NEGR1 has enhanced expression in the brain and may be involved in regenerative axon sprouting [24]. Further, NEGR1 protein expression is not detected in the human cervix, and so we did not pursue these pathways further.

On the other side of the ledger, of the four pathways repressed significantly by siE6/E7, three were still repressed upon co-depletion of Set8 (albeit, without statistical significance in the case of E2F targets, as described above). The Myc targets V1 pathway was the only pathway that was repressed by siE6/E7 (when Set8 is elevated) and activated by co-depletion of Set8 (Supplementary Figure S2A,B). Thus, it is possible that Myc targets V1 pathway genes are repressed by Set8, and the degradation of Set8 by E6 is important for the upregulation of these genes in HPV transformed cells. However, there was no individual gene in the pathway that showed reproducible and significant repression upon siE6/E7 and activation by siE6/E7+siSet8.

These results suggest that disruption of Set8 mediated gene regulation by HPVE6 may be collectively important for the repression of myogenesis and apical junction pathways, and the activation of Myc targets V1 pathway in HPV transformed cells, but it is difficult to attribute the change to a few key genes in these pathways.

**Figure 3:**
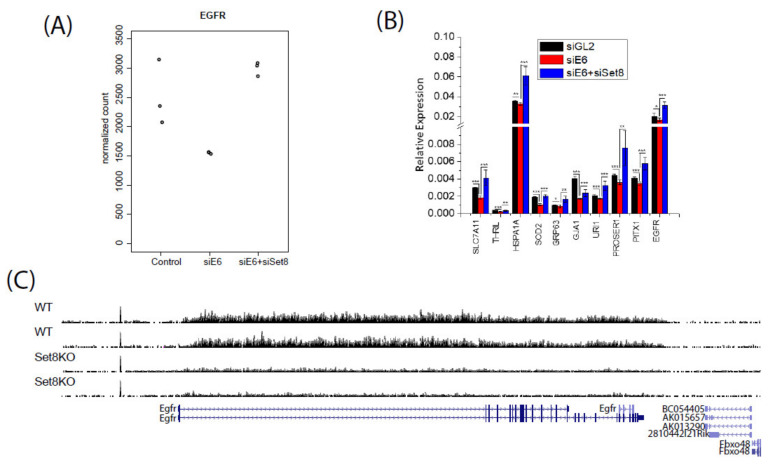

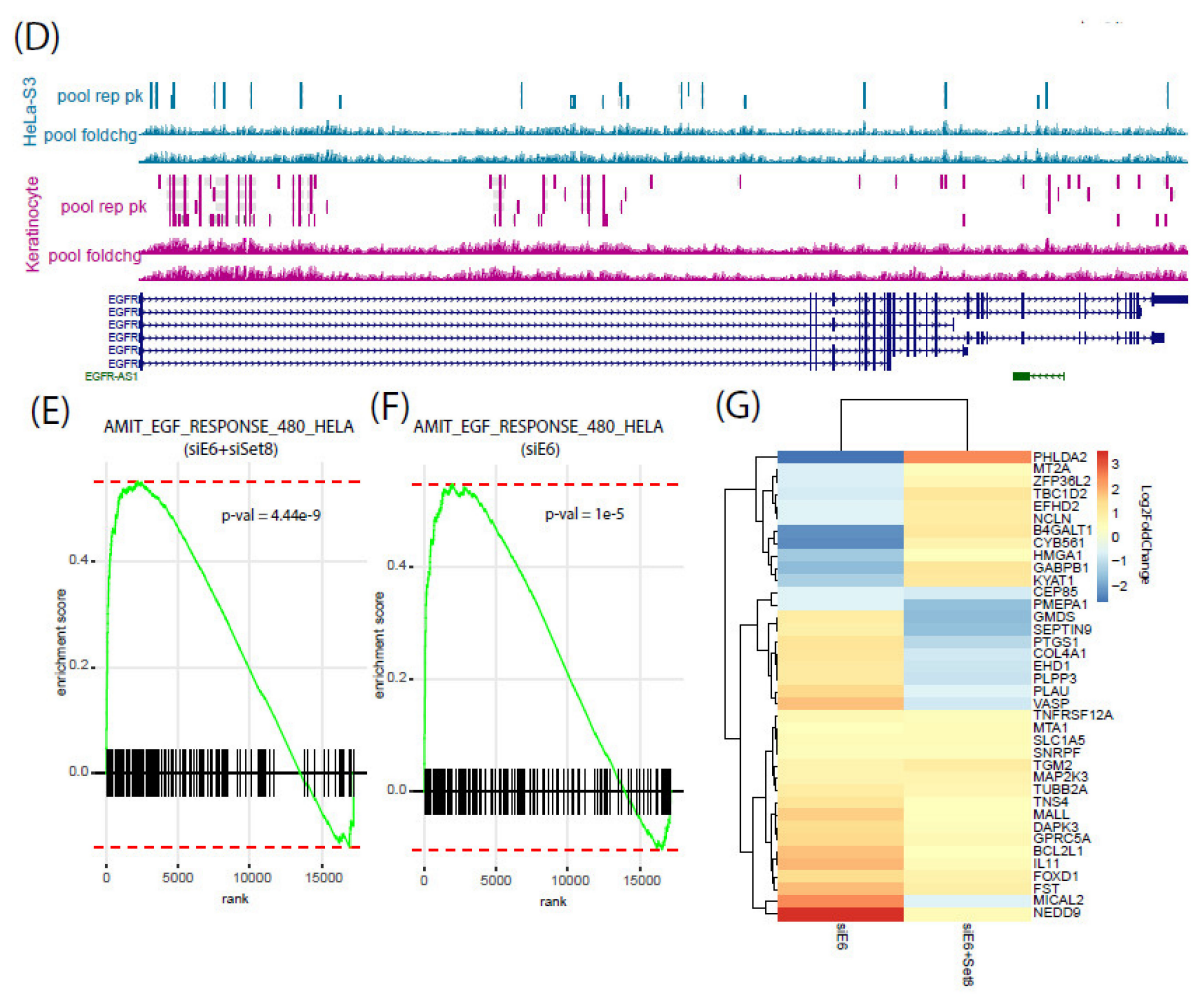
E6 induces EGFR expression via degradation of Set8 and controls EGF response genes. (**A**) EGFR expression in siE6 and siE6+siSet8 conditions from RNA sequencing. (**B**) qPCR validation of RNA sequencing results on ten genes in HeLa cells that were repressed by siE6 and where the repression was rescued by co-knockdown of Set8 * *p* < 0.05, ** *p* < 0.001, *** *p* < 0.0001. (**C**) H4K20me1 ChIP-seq reads in the EGFR gene from liver specific Set8 knockout mice (GSE97338) compared to that in WT mice. (**D**) ENCODE H4K20me1 ChIP-seq peaks in the EGFR gene from HeLa-S3 (HPV+) and keratinocytes. (**E**) and (**F**) EGF response gene set enrichment analysis for siE6+siSet8 relative to control (siGL2) and siE6 relative to control, respectively. (**G**) Heatmap of log2 fold change of highly expressed genes within the EGF response gene set from (**F**) and (**G**).

We therefore turned our attention to changes in expression of ten individual genes that may have a role in oncogenesis in a pattern that suggested they may be regulated by E6 through Set8: change in expression by siE6 is reversed by co-knockdown of Set8. For example, E6 knockdown reduces EGFR expression while E6 and Set8 knockdown rescues EGFR expression in the RNAseq data (Figure 3A). This suggests that E6 induces the expression of EGFR via degradation of Set8. The RNA sequencing results were validated in an independent experiment by qPCR for EGFR and nine other highly expressed genes that were repressed by siE6/E7 and where the repression was rescued by siSET8 (Figure 3B). The changes were subtle, consistent with the small changes in H4K20me1 level that we saw in the transient knockdown experiments (Figure 2A).

In order to determine if long term Set8 depletion alters H4K20me1 at the EGFR gene, we compared publicly available H4K20me1 ChIP-seq data in wild type and Set8 knockout mouse liver [25,26]. H4K20me1 methylation across the EGFR gene was reduced in the Set8 knockout mice (Figure 3C). Thus, Set8 is responsible for this modification on the EGFR gene. To determine whether E6, by promoting the degradation of Set8, decreases the H4K20me1 modification on the EGFR gene, we compared H4K20me1 ChIP-seq profile in the HPV transformed HeLa cells to that in normal keratinocytes from ENCODE [26]. Indeed, there were fewer H4K20me1 peaks in HeLa cells compared to keratinocytes, suggesting that E6 decreases this repressive mark on the EGFR gene (Figure 3D).

The ChIP-seq and RNA expression results are consistent with the hypothesis that E6 upregulates EGFR through the degradation of Set8, a writer of repressive histone marks on the EGFR gene. We thus expected that EGF-responsive gene will be repressed by siE6, and the repression will be rescued by co-knockdown of Set8. However, the AMIT_EGF_RESPONSE_480_HELA gene set [27] in the RNA sequencing data revealed that EGF responsive genes are mostly induced upon E6 knockdown (Figure 3F) and this continues after E6 and Set8 knockdown (Figure 3E). Thus the epigenetic activation of EGFR in E6 transformed cells does not automatically lead to the up-regulation of all the genes in the EGF induced gene set (which would have led to repression of the genes after siE6). We therefore hypothesized that specific genes in the EGF-induced set were differentially regulated by E6 through Set8. Consistent with this, there were several genes in the EGF response dataset that were either increased by E6 knockdown and decreased by E6+Set8 knockdown and vice versa (Figure 3G). Thus although the EGF responsive genes appear to be regulated by E6, the exact direction of change in expression of the EGF responsive genes cannot all be explained by a simple induction of EGFR through the degradation of Set8 by E6/E7.

Taken together these data suggest that the E6 mediated degradation of Set8 contributes to the alterations of the epigenome and gene expression seen in HPV transformed cancers.

### 2.3. Recombinant HPV-E6 Activates Enzymatic Activity of USP46 In-Vitro

**Figure 4:**
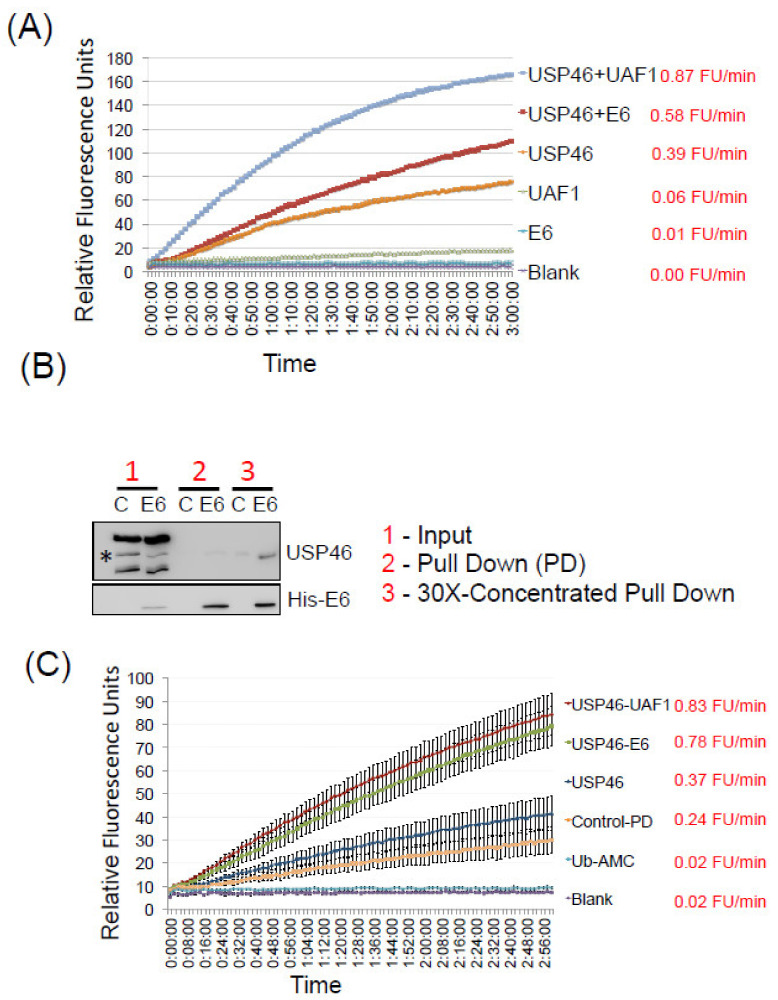
(**A**) Ub-AMC deubiquitination assay: Fluorescence intensity of 7-amido-4-methylcoumarin (AMC) fluorophore released from Ub-AMC substrate indicating the deubiquitinase activity of indicated proteins. Marker indicates 1-min time point measurements for 3 h after start of the reaction. Ub-AMC alone was used as the blank input. (**B**) Untagged USP46 expressing Sf9 cell lysates was mixed with His-E6 protein overnight followed by purification of the complex on Ni-NTA columns. Purified complex was concentrated 30 times. Input, purified and concentrated (30×) complex were immunoblotted with indicated antibodies. (**C**) Deubiquitination assay as in (**A**), with the purified complex.

USP46 is enzymatically inactive by itself and requires co-factors like UAF1 or WDR20 to exhibit its enzymatic activity. In our previous report we demonstrated that the E6:USP46 complex did not contain UAF1 or WDR20. In addition, E6 immunoprecipitates could activate the enzymatic activity of recombinant USP46, but it was unclear whether the immunoprecipitate brought other cellular factors into the reaction. Hence, we wanted to test if recombinant E6 can induce the enzymatic activity of USP46 in vitro. For this we purified recombinant HPV-E6 and USP46 from bacterial and insect cells, respectively. The co-factor human UAF1 was also purified from insect cells as a positive control for induction of USP46 activity. Although insects contain homologs of UAF1 and WDR20 (e.g., *Drosophila* genes CG9062 and CG6420, respectively), Coomassie staining of the purified USP46 protein showed that it was not stoichiometrically associated with UAF1 or WDR20 (Supplementary Figure S3). Consistent with this, the recombinant USP46 was not very active by itself: 0.39 fluorescence units per minute (FU/minute) towards an artificial substrate ubiquitin–aminoluciferin (Ub-AML). Addition of the UAF1 to the recombinant USP46 resulted in a two-fold increase in the deubiquitinase activity of USP46 (0.87 FU/min), also consistent with the conclusion that the recombinant USP46 was free of UAF1 or WDR20. Addition of recombinant HPV-E6 increased the activity by 1.15 fold to 0.58 FU/min (Figure 4A). This suggested that HPV-E6 could be added to recombinant USP46, even when UAF1 was absent, to stimulate the deubiquitinase activity.

The increase in the activity mediated by HPV-E6 was less compared to UAF1, making us wonder whether this was because most of the USP46 was not complexed with E6 when the two were simply mixed in vitro. To determine the extent of the association, we expressed untagged USP46 in insect cells and incubated the lysate with recombinant His-E6. Purification of the His-E6:USP46 complex on Ni-NTA column revealed that only a minor pool of the expressed USP46 bound to E6 and could be seen, and that too only upon concentrating the pulled-down material 30X (the USP46 band denoted by * in Figure 4B). The purified E6:USP46 complex (where all the USP46 had to be associated with E6) was concentrated and applied to in-vitro deubiquitination of Ub-AMC substrate. The purified complex displayed a deubiquitinase activity similar to the USP46-UAF1 complex. The activity of purified E6-USP46 complex was 0.78 FU/min compared to 0.83 FU/min for USP46-UAF1 complex (Figure 4C). Therefore E6 alone can substitute for UAF1 in inducing the catalytic activity of USP46.

### 2.4. Multiple Domains of Cdt2 Protein Are Required for Its stabilization by E6-USP46 Complex

**Figure 5:**
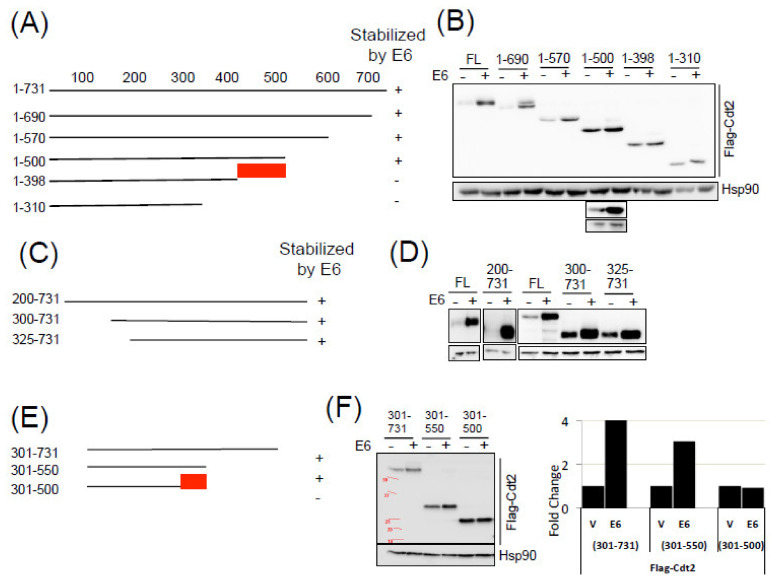

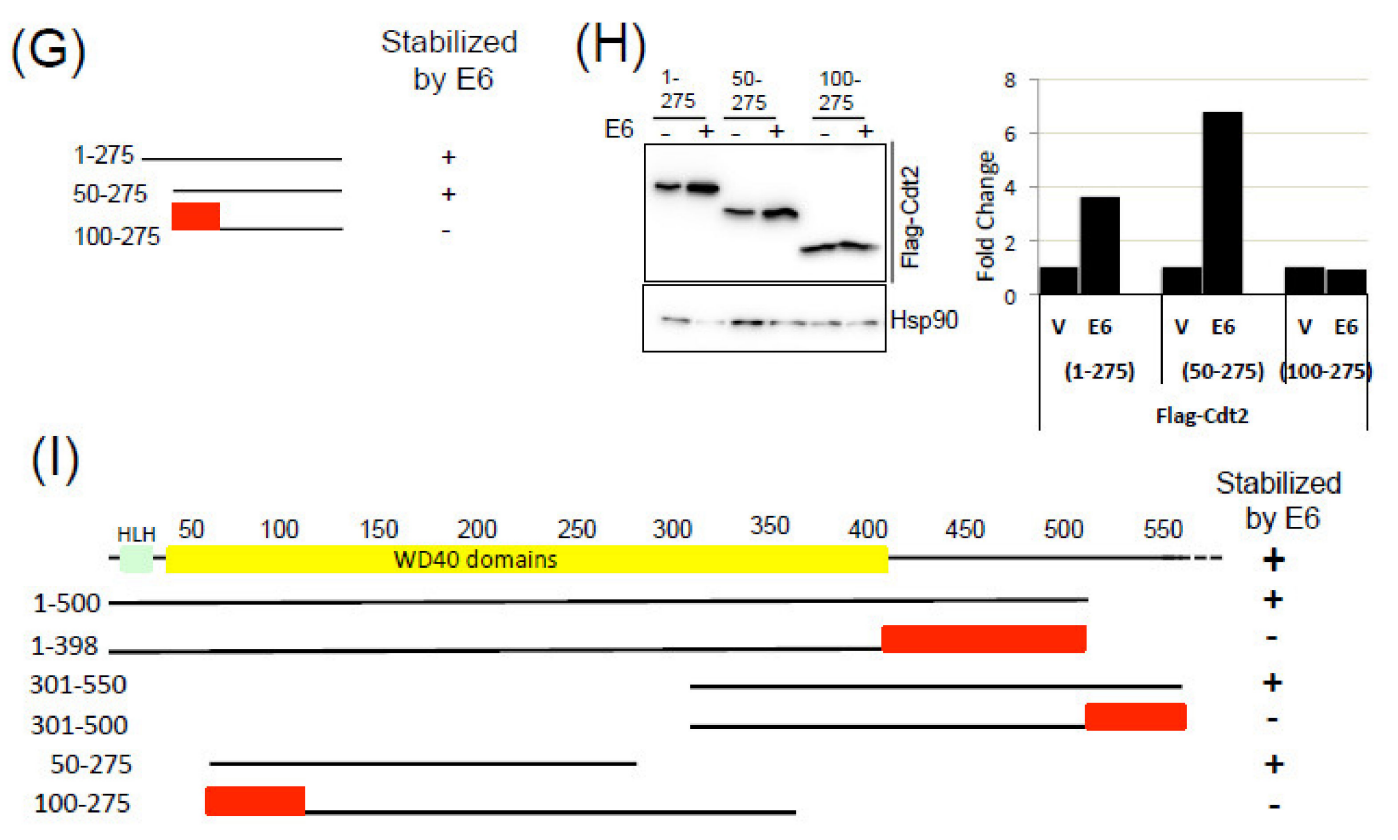
Schematic on the left (**A**,**C**,**E**,**G**) indicate Cdt2 sequences being tested for stabilization by HPV-E6 on the right. (**B**,**D**,**F**,**H**)- Indicated Cdt2 deletion constructs were transfected in U2OS cells with control or HPV-E6 expressing vectors and cells were collected 48 h after transfection. Cell lysates were then immunoblotted with indicated antibodies. A separate set of results is presented for Cdt2 1–500 construct to show its robust stabilization by E6. (**I**) Schematic summarizing the critical deletions that identified the sequences that promote stabilization of Cdt2 by E6 relative to the locations of the HLH domain (interacts with DDB1) and the WD40 domain. Red boxes: regions necessary to see the stabilization in the various deletion constructs.

In our previous paper, we have demonstrated the interaction of Cdt2 with the E6:USP46 complex and the stabilization of Cdt2 by the complex, but the Cdt2 sequences required for the interaction/stabilization were not identified. To identify these sequences, N- and C- terminal Cdt2 deletion constructs were transfected into cells and checked for their stabilization by the co-transfection of exogenous HPV-E6. C- terminal sequential deletions demonstrated the presence of a stabilization signal between residues 398 to 500 of Cdt2 because a construct encoding 1–500 amino acids of Cdt2 was stabilized by HPV-E6 but not a construct encoding 1–398 amino acids of Cdt2 (Figure 5A,B). N-terminal deletion constructs encoding 200–731, 300–731 and 325–731 amino acids were all stabilized by E6 (Figure 5C,D). Deletion of C-terminal ends on construct encoding 301–731 amino acids, revealed the importance of residues 500–550, as 301–550 amino acids was stabilized while 301–500 was not stabilized by HPV-E6 (Figure 5E,F).

Comparing the stabilization of 1–500 with the failure to stabilize 301–500 suggested that a signal in the N-terminal 300 residues might also be important for stabilization. To investigate this, a construct encoding 1–275 amino acids was tested and found to be stabilized by HPV-E6 (Figure 5G,H) indicating the presence of stabilization signals in the N terminus of Cdt1. Sequential deletions from the N terminus in the 1–275 construct revealed that a sequence between 50 and 100 amino acids is important for stabilization because a construct encoding 50–275 amino acids of Cdt2 was stabilized, while that encoding 100–275 amino acids was not (Figure 5G,H). Here again, the stabilization of 200–731, 300–731 or 325–731 suggests that the N terminal 50–100 sequences for stabilization is redundant with the 500–550 region, and its importance is not seen when the latter sequence is present. Thus, we identified at least three sequence stretches that are important for E6-USP46 mediated stabilization of Cdt2: 50–100, 398–500 and 500–550 amino acids, with redundancy between the N terminal 50–100 and the C terminal 500–550 signals, while the middle signal (398–500) cannot seem to stabilize without at least one of the two other signals.

## 3. Discussion

Activities of the E6 oncoprotein in HPV-induced cancers are essential for maintenance of the oncogenic state of HPV-induced cancers [28]. The viral protein redirects several host proteins to maintain a high proliferative state. Accordingly, E6-interacting proteins such as the E3 ubiquitin ligase, E6AP, are seen as attractive targets for treatment of such cancers [29]. We have identified USP46 as one of the effector proteins that induces stabilization of Cdt2 oncoprotein through interaction with HPV E6. The loss of Set8 protein along with the loss of H4K20 mono and tri methylation marks in HPV-induced cancer biopsies indicates that E6-USP46-Cdt2-Set8-H4K20me1 signal transduction pathway is active in actual cancers to decrease the repressive mark on the genome. Paradoxically, the change in histone methylation was not as marked after acute alteration of Cdt2 and Set8 over 1–2 cell-cycles indicating that its chronic alteration in cancers in vivo is necessary to effect the observed changes in the global epigenetic landscape of HPV-infected cancers.

Because the in vitro increase in H4K20me1 after E6 depletion and Set8 restoration for up to 48 hr in HPV positive cancer cells was not significant, we did not expect to see large-scale changes in gene expression under those conditions. However, it was possible that individual loci regulated by H4K20me1 may be more responsive to the transient increase in Set8 following E6 depletion. By comparing gene expression profiles of cells where Set8 and E6/E7 were co-depleted versus cells where only E6/E7 was depleted we found a handful of genes whose regulation was consistent with them being regulated by the E6-USP46-Set8 axis in the timescale of the assay. Gene set enrichment analyses (GSEA) can identify minor but consistent changes in gene expression patterns integrated over multiple genes in the set. There were some hints that gene sets related to apical junction and myogenesis were activated by Set8 (and so repressed in E6 expressing cells), while gene sets that were Myc targets were repressed by Set8 (and so activated in E6 transformed cells), but there were no suitable individual genes in these gene sets that could be studied in detail.

Examining individual genes independent of gene sets identified ten genes that could be repressed by Set8, and so were induced by E6 through the destruction of Set8. Of these, the EGFR gene was most promising because there was evidence of H4K20me1 marks at this gene locus being repressed by Set8 and de-repressed by E6 consistent with the hypothesis that E6 can regulate at least some genes by altering Set8 levels even in transient experiments. EGFR is known to be up-regulated in 40–50% of cervical squamous cell carcinomas [26,27,28]. Enhanced EGFR expression is a feature of aggressive cancers and significantly lowers the prognosis of survival [30,31]. Inhibition of EGFR signaling significantly impedes growth of cervical tumors [32]. EGFR activating mutations are not present in cervical carcinomas [31,32], so that induction of the gene by epigenetic pathways seems a plausible approach to increasing its activity in such carcinomas. Another HPV oncoprotein, E5, increases EGFR protein by stabilizing it through inhibition of its ubiquitination [33,34]. However, we are seeing changes in the EGFR RNA level, so E5 could not be contributing in any way to our results. Thus, HPV E6 has an independent role in upregulating EGFR by removing repressive marks on the locus through the E6-USP46-Cdt2-Set8 pathway. It is likely that we did not see a consistent upregulation of EGF target genes in these experiments because the viral E5 protein was absent to stabilize the EGFR protein. Alternatively, the cell culture conditions are likely to be replete with growth factors that diminish the importance of levels of EGFR in regulating gene expression and cell proliferation.

Together with the decrease of H4K20me1 and H4K20me3 modifications in cervical cancer biopsies, the results described above provide evidence that the E6-USP46-Cdt1-Set8 axis can contribute to epigenetic and gene expression changes seen in HPV-positive cervical cancers. Given the minor changes in H4K20me1 levels seen globally in the transient in vitro experiments, finding the complete list of genes that are regulated through this axis should ideally use in vivo experiments in xenografts of HPV-transformed cancers. However, E6 depletion experiments similar to those carried out here, which are essential to prove causality, will be difficult because (a) the xenografts do not survive after E6 depletion and (b) long term Set8 depletion or over-expression is toxic for cells.

A critical observation in our previous paper was that the E6:USP46 complex lacked UAF1 or WDR20 [15]. Normally UAF1 and WDR20 bind to the finger-domain and the base of the palm domain of USP46, away from the catalytic site, and induce essential conformational changes around the catalytic center of USP46 that contribute to the catalytic activation [19]. The activation of recombinant USP46 deubiquitinase by recombinant HPV-E6 suggests that E6-USP46 complex also stabilizes the conformational changes in the catalytic center necessary for activation of the deubiquitinase. It was noteworthy, that contrary to what we see in cells, where USP46:E6 association is easily detected, only a minor fraction of the recombinant USP46 and E6 was found to associate with each other *in vitro*. Therefore, a specific modification on either protein or a cellular or viral chaperone may be necessary for the formation of the complex, but once formed, such an E6:USP46 complex is stable and catalytically active. These results are important for future efforts to identify chemical inhibitors of the active USP46, which are predicted by our experiments to have an anti-proliferative effect specifically in HPV transformed cancers [15].

We and others have shown that Cdt2 is degraded by ubiquitination by specific E3 ubiquitin ligases [15,35], making it plausible that the deubiquitination of Cdt2 by E6:USP46 leads to the stabilization of Cdt2. Our identification here of specific regions on the substrate (Cdt2) necessary for its stabilization by HPV E6 now provides further support for this hypothesis. Stabilization of Cdt2 by E6:USP46 needs three prerequisites: (a) interaction of Cdt2 with E6:USP46, (b) recognition of Cdt2 by its E3 ubiquitin ligases and (c) the presence on Cdt2 of sites of polyubiquitination. Therefore, it is not surprising that instead of one region, we identified at least three different regions of the protein as being important for the stabilization (summarized in Figure 5I). We know that all three regions are not required for stabilization, because at least one of the stabilized deletion constructs, 1–275, contained only the 50–100 region. In addition, as discussed in the results, there is redundancy between the N terminal 50–100 and the C terminal 500–550 signals in their ability to support stabilization in the presence of the middle 398–500 region.

We have shown that USP46 co-immunoprecipitated from cells expressing E6 contain Cdt2 complexed with DDB1 [15], and so we wondered whether the interaction with USP46 requires the presence of DDB1. The structure of Cdt2 has not been solved, but that of related proteins, DCAFs like DDB2 or CSA, in complex with DDB1 of the CRL4 complex have been solved [36]. The N terminus of Cdt2 (residues 3–26) has a helix–loop–helix (HLH) motif that has been proposed (by similarity to CSA) to interact with DDB1, but this region is dispensable for the stabilization of Cdt2 (e.g., 50–275, 325–731 or 301–550) (Figure 5I). Furthermore residues 50–398 of Cdt2 contain seven WD40 repeats that together are predicted to form a beta-propeller that is critical for the structural integrity of the protein and its interaction with DDB1 or with the substrate of CRL4-Cdt2 (Figure 5I). Many deletions that disrupt the beta propeller (e.g., 200–731, 300–731, 325–731, 1–275) are still stabilized by E6, suggesting that the beta propeller and the structural integrity of Cdt2 are not important for USP46 to interact with and deubiquitinate the protein. Therefore although our co-immunoprecipitation experiments showed that DDB1 is present in the USP46:Cdt2 complex [15], neither DDB1 nor a substrate of the CRL4-Cdt2 complex is required for the E6:USP46:Cdt2 interaction and subsequent Cdt2 stabilization

## 4. Materials and Methods

### 4.1. Cell Culture

HeLa and U2OS cells were cultured in Dulbecco’s modified Eagle’s medium (DMEM) containing 10% fetal bovine serum (FBS). They were grown in humidified incubators with 5% CO2. The cell lines were authenticated by analyzing 15 short tandem repeats and the amelogenin gene at Biosythesis (https://www.biosyn.com/index.aspx, accessed on 12 August 2021).

### 4.2. Cell Cycle Synchronization following HPV-E6 Depletion and Overexpression

For HPV-E6 depletion, 10 nM of siRNAs for GL2 (siGL2–5′-CGTACGCGGAATACTTCGA-3′) or HPV18-E6 (si18E6- 5′-CAGAGAAACACAAGTATAA-3′) were used to transfect 3 × 10^6^ HeLa cells by reverse transfection. A 2nd transfection was done 24 h later. 48 h after first transfection cells were treated with 2 mM Thymidine for 14 h followed by its removal and washing of cells by PBS. 9 h later 2 mM Thymidine was again added for 14 h followed by its removal, PBS wash and addition of fresh media. Various time points were collected thereafter.

For HPV-E6 overexpression, 4 μg of plasmids p1322-E6 (Addgene # 8642) or p1322-GFP (lab generated) were transfected in 2 × 10^6^ HeLa. Lipofectmine-3000 was used as the transfection reagent as per the manufacturer’s protocol. pBABE-puro was added in 1/10th molar ratio to allow for puromycin selection of transfected cells. 24 h after transfection, puromycin was added for 24 h to select for transfected cells. 48 h after transfection 2 mM Thymidine was added for 14 h followed by removal and washing of cells by PBS. 9 h later 2 mM Thymidine was again added for 14 h followed by removal and PBS wash. Fresh media was added and various time points were collected, thereafter.

### 4.3. Immunohistochemistry

Tissue sections were cut from each block at 4 mm thick intervals. Antigen retrieval and deparaffinization were performed in PT Link (Dako, Glostrup, Denmark) using high pH EnVison FLEX Retrieval solution (Dako) for 20 min at 97 °C. Immunohistochemistry was performed on a robotic platform (Autostainer, Dako). Endogenous peroxidases were blocked with peroxidase and alkaline Phosphatase blocking reagent (Dako) before incubating the sections with respective antibodies. Antibodies used were Set8 (CST #2996), H4K20-me1(AbCam #ab9051) and H4K20-me3 (active motif #07-463). Antigen-antibody complex was detected using DAKO Envision, anti-rabbit polymer followed by incubation with 3,30-diaminobenzidine tetrahydrochloride (DAB+) chromagen (Dako). All the slides were counterstained with hematoxylin subsequently; they were dehydrated, cleared and mounted for the assessment.

The images were captured and 150 cells from normal and cancerous areas were randomly scored from 1 to 4 based on staining intensity. The staining score measures the proportion of cells with highest score (4). For example, if A, B, C and D number of cells were given scores of 4, 3, 2 and 1, respectively, then the staining score is (4 × A)/[(1× D) + (2 × C) + (3 × B) + (4 × A)].

### 4.4. Immunoblotting

For immunoblotting cell lysates were prepared in NP40 lysis buffer (Tris-Cl, 50 mM, pH8; NaCl-150mM and 1% NP40). Lysates were subjected to SDS-polyacrylamide gel electrophoresis followed by transfer to nitrocellulose membrane. The blots were probed with required antibodies To avoid re-probing a blot too many times and because some of the proteins required different acrylamide gel concentrations, a few of the blots were run separately from others in the panel.

### 4.5. Purification of the Recombinant E6, USP46 and UAF1 Proteins

HPV18-E6 (4C-4S) version of E6 fused with His-tag was expressed in bacterial *E. Coli* BL21 cells at 15 °C for 20 h. The plasmid expressing His-E6 in bacteria was a gift from Shreya Dharadar and Titia Sixma (Netherlands Cancer Institute, Amsterdam, the Netherlands). The His-E6 protein was purified on Ni-NTA columns by affinity chromatography.

His-USP46 or untagged USP46 was cloned in pFastbac-HTC vector and then transformed in DH10Bac cells. Blue-white selection and PCR screening identified DH10Bac clones having recombinant bacmids with USP46. Bacmid was purified and transfected in adherent Sf9 cells and death of Sf9 cells was used to signify successful generation of baculovirus particles for expression of USP46. Supernatant from such cultures containing was collected and virus concentration was amplified by adding it to Sf9 cells for two more passages (P1 and P2). P2 virus was used to transfect Sf9 cells in suspension to express USP46 for 48 h. Cells were pelleted and lysed and His-USP46 was purified on Ni-NTA columns by affinity chromatography.

### 4.6. Deubiqutination Assay

To assess the deubiquitinase activity of the E6-USP46 complex, Ub-AMC was used as an artificial substrate and release of α–methyl-coumarin (AMC) was used as indicator of the deubiquitinase activity. 70 nM of USP46 and 4 μM of E6 and 5 μM of Ub-AMC were mixed in 14 μL of deubiquitination buffer (PBS with 0.02% Tween-20, 1 mM TCEP, and 0.1 mg/mL human serum albumin). The reaction was carried out in 384 well plates and release of AMC was monitored by measuring fluorescence on a plate reader at excitation and emission wavelength of 345 and 445 nm respectively (Figure 4A). 400 nM of UAF1 was added as a positive reaction for activation of USP46. For the experiment in Figure 4A the singly purified proteins were mixed in vitro in 384 well plates. The assay was started immediately after mixing the proteins in 384 well plates.

For purifying E6-USP46 complex for deubiquitination reaction (Figure 4B,C), untagged USP46 was expressed in Sf9 cells and cell pellet from 100 mL culture was lysed in 200 mL of lysis buffer (50 mM Tris pH 7.5, 300 mM NaCl and 0.1% Triton X-100). 500 μg of recombinant His-E6 was then added to 50 mL USP46 expressing lysate and allowed to incubate overnight at 4 °C. After that Ni-NTA beads (Qiagen) were added and incubated for 60 min. Lysates from cells not expressing (control) or expressing untagged USP46 were then transferred to the columns and the bound complex was washed with lysis buffer six times to remove proteins binding non-specifically. The proteins bound to His-E6 were then eluted in 4 washes of 150 μL of elution buffer (50 mM Tris pH 7.5, 150 mM NaCl, 250 mM Imidazole). 450 μL of the eluate was concentrated to 15 μL volume in Microcon-30 kDa Centrifugal Filter Unit with Ultracel-30 membrane and 2.5 μL was used for the deubiquitination reaction with Ub-AMC substrate as above. The USP46 in the reactions were equalized by estimating the USP46 quantity by gel electrophoresis and immunoblotting in the three preparations containing USP46.

### 4.7. Cdt2 Deletion Constructs

Full length Flag-Cdt2 cloned in pEF vector was used as a template for various deletions in Cdt2 [37]. Inverse PCR primers were designed across sequences desired for deletion as described previously [30]. These plasmids were transfected in U2OS cells with control vector or HPV-16E6 using Lipofectamine-LTX as per manufacturer’s instructions. 0.1 million cells were transfected in 12 well plates. Cells were collected 48 h after transfection and collected for western blotting.

### 4.8. Total RNA Isolation, cDNA Synthesis and qPCR

Total RNA isolation was carried out using RNeasy mini kit (Qiagen) after 48 h from HeLa cells transfected with control siRNAs or HPV-E6 siRNA or HPV-E6 and Set8 siRNAs. 1 μg of total RNA was reverse transcribed using SuperScript III First-Strand cDNA synthesis kit (Thermo Fisher Scientific, Waltham, MA, USA, catalog no. 18080051). QPCR was performed using Power SYBR green master mix reagent (Thermo Fisher Scientific, catalog no. 4367659) on a Step One plus qPCR machine (StepOne Plus Thermo Fisher Scientific) and fold induction was calculated using the ΔΔCt method. The fold induction was normalized against loading control GAPDH. The Q-RT-PCR was done on 3 replicates.

### 4.9. RNA Seq Sample and Library Preparation

HeLa cells were transfected with control siRNAs or HPV-E6 siRNA or HPV-E6 and Set8 siRNAs. Cells were collected 48 h after siRNA transfection and RNA was extracted using the Qiagen RNA extraction kit. RNA samples were sent to Beijing Novogene Co., Ltd. (Beijing, China) for library preparation and next generation RNA sequencing. More than 50 million paired end reads were obtained for each sample. Three experimental replicates were prepared for each sample and RNA integrity numbers (RIN) derived from a Bioanalyzer were used to ascertain the quality of RNA.

### 4.10. mRNA Sequencing Analysis

Paired end mRNA sequencing fastq files were mapped to the human genome version GRCh38 using kallisto 0.44.0 [38]. Abundance.h5 files were imported into R using tximport [39] and differential expression analysis conducted using DESeq2 [40]. fgsea package was used for gene set enrichment analysis, with genes ranked by the Wald statistic from DESeq2 analysis [41].

### 4.11. Chromatin Immunoprecipitation Sequencing Analysis

H4K20me1 ChIP-seq bigwig files were downloaded from GEO accession GSE97338. HeLa-S3 and keratinocyte H4K20me1 ChIP-seq files were obtained from the ENCODE consortium. Bigwig and peak files were visualized using the UCSC genome browser.

## 5. Conclusions

**Figure 6:**
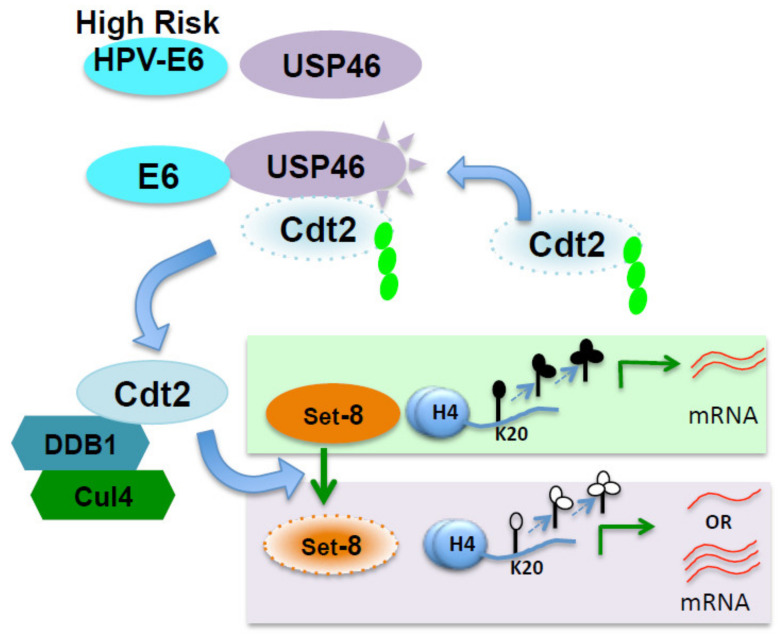
Model summarizing the role of the E6-USP46 complex in HPV induced cancers: E6 from high-risk HPVs engage with and activate the deubiquitinase activity of USP46. The complex then interacts with and stabilizes Cdt2, which in turn degrades the H4K20 mono-methyl-transferase Set8 resulting in decreased levels of H4K20 mono, di and tri methylations at H4K20. Depending on the gene this results in up or down regulation of several genes (like EGFR).

In conclusion, we find that the HPV E6 protein uses the E6-USP46-Cdt2-Set8 pathway to effect epigenetic and gene expression changes in HPV-induced cervical cancers and cell line (Figure 6), that the E6 substitutes for the critical role of UAF1 in activating the USP46 deubiquitinase, and that specific regions of Cdt2 are necessary for its stabilization by E6-USP46, ruling out non-specific modes by which Cdt2 might have been deubiquitinated and stabilized. These findings set the stage for screening of inhibitors of USP46 (particularly E6:USP46) that could be of therapeutic value in HPV-induced cancers.

## Data Availability

The RNA sequencing data generated in this study is publicly available. This data can be found here: the NCBI Gene Expression Omnibus (GSE191010).

## Supplementary Materials

The following are available online at https://www.mdpi.com/article/10.3390/cancers14010030/s1, Figure S1: (A) Specificity of Set8 antibody checked by immunostaining formalin-fixed paraffin embedded DM93 melanoma cells and genomically partially deleted Set8 in DM93 cells [42]. (B) Ki67 staining of cancerous and adjacent normal cervical tissues used in Figure-1A. Figure S2: Summary of Geneset enrichment analysis (GSEA) following RNA sequencing. Hallmark pathways were tested for enrichment (up regulated: bars to the right) or disenrichment (down regulated: bars to the left) in these two conditions. significance of the change after adjusting for multiple hypothesis testing (Benjamini-Hochberg method) is indicated by the colors indicated in the key. Figure S3: Coomassie staining of the recombinant USP46 shows that it is not stoichiometrically associated with any insect protein. Dilutions of BSA were used for accurate quantitation of the recombinant USP46. Figure S4: Original western blots. Tables S1: Metrics indicating quality of RNA sequencing. Table S2: Metrics summarizing the alignment of RNA sequencing reads.
