# Supplementary material for: HPVE6-USP46 Mediated Cdt2 Stabilization Reduces Set8 Mediated H4K20-Methylation to Induce Gene Expression Changes
Source: Cancers (Basel). 2021 Dec 22;14(1):30. doi: 10.3390/cancers14010030 (PMC8750077; doi:10.3390/cancers14010030)
Supplement: Supplementary file 1 [file cancers-14-00030-s001.zip › cancers-1433038-supplementary.pdf]

Supplementary -1

(A) Set8 antibody specificity

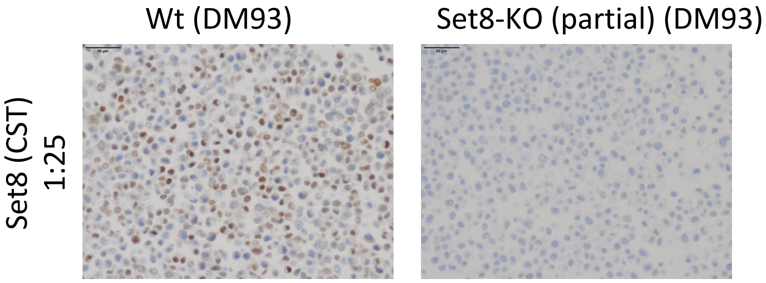

(B) Ki67 staining pattern in normal and cancerous pairs

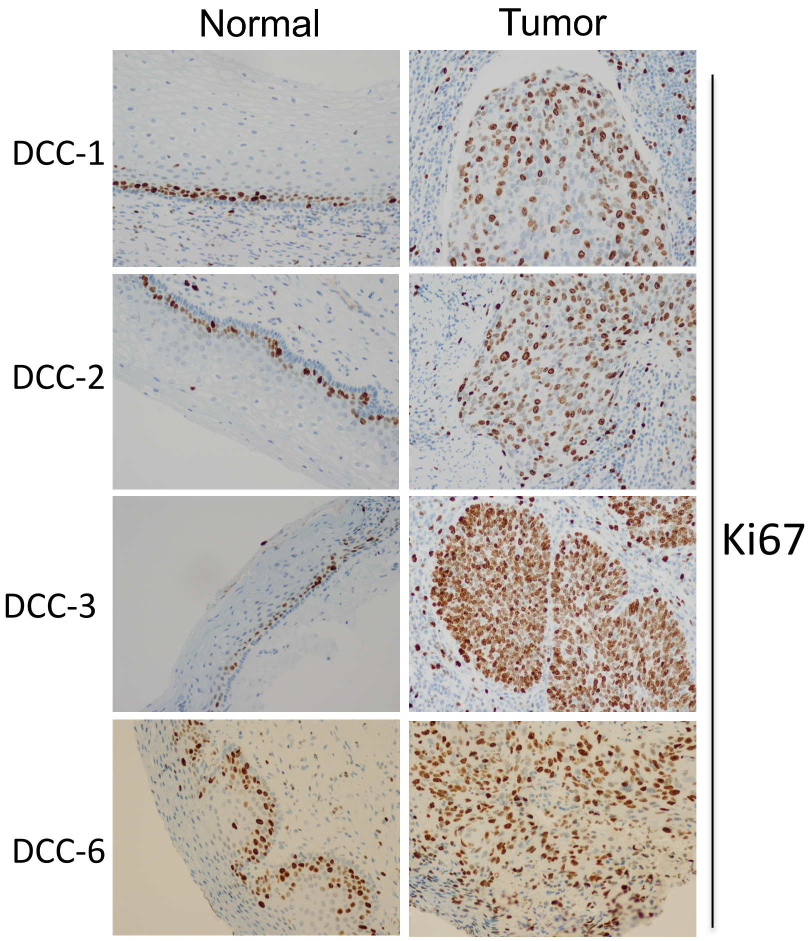

Supplementary-2

(A)

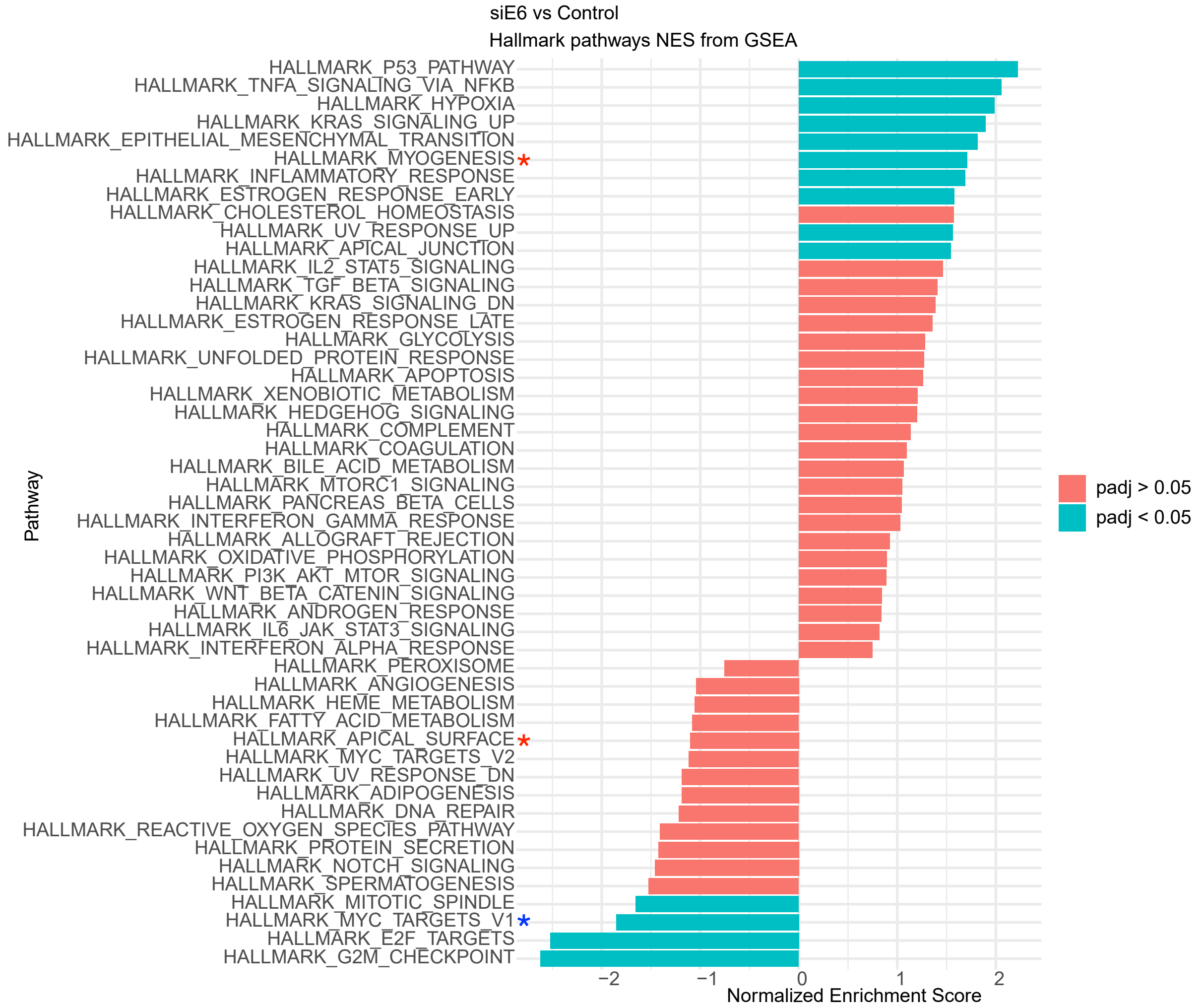

(B)

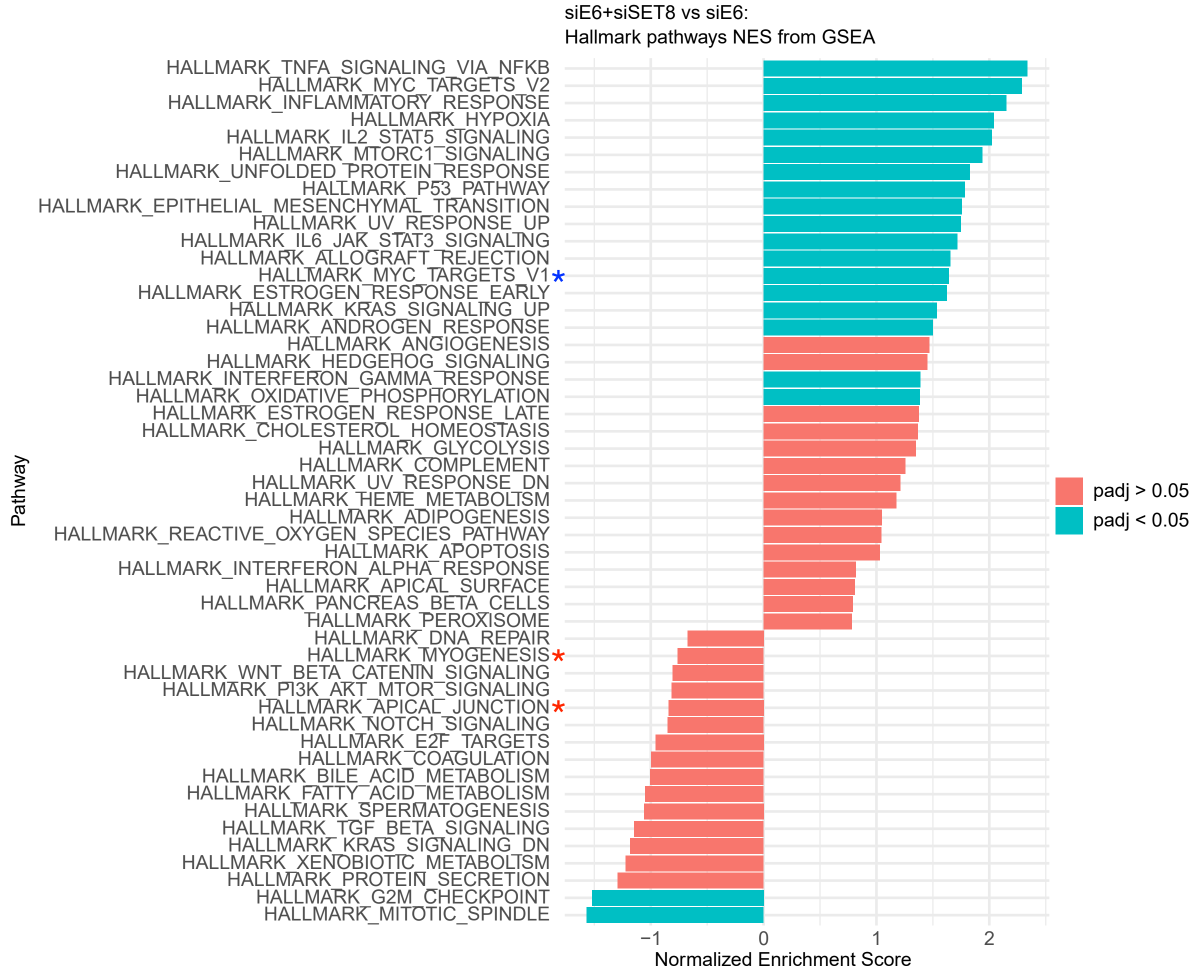

Supplementary-3

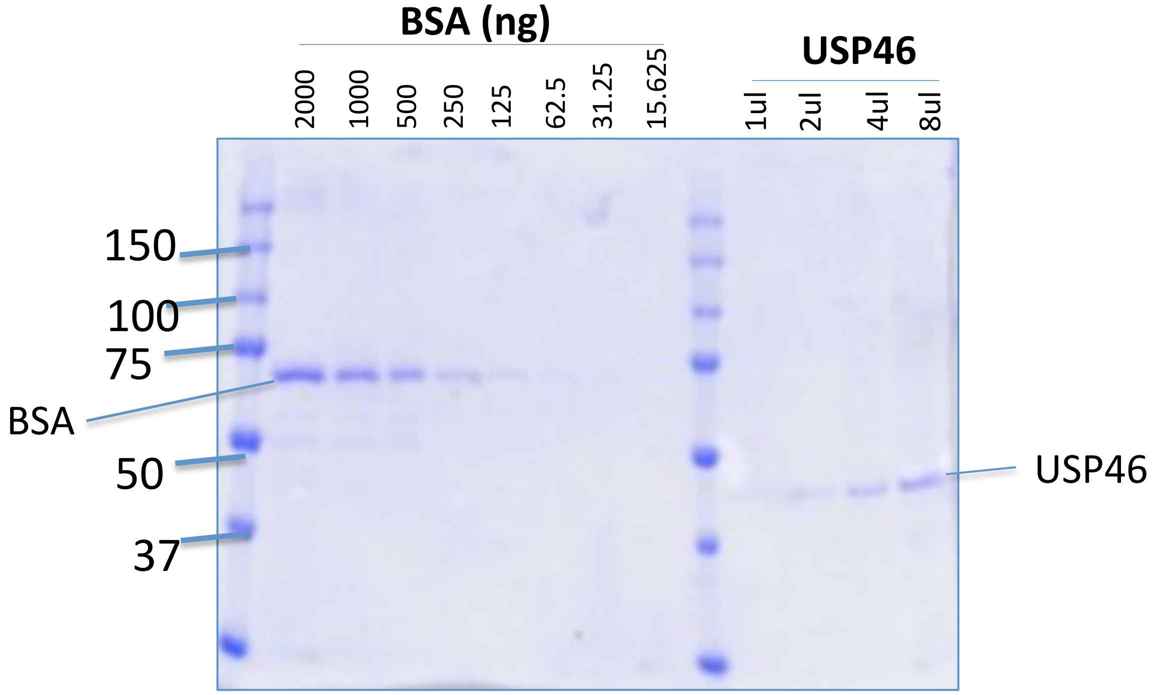

Supplementary Tables

(S1) Data table

Sample name	Raw reads	Clean reads	Raw bases	Clean bases	Error rate(%	Q20(%)	Q30(%)	GC content(%)
SK01 (Gl2-Contro)	71130612	70026436	10.7G	10.5G	0.03	97.86	94.16	51.31
SK02 (siE6)	73431482	72178864	11G	10.8G	0.03	97.8	94.01	51.55
SK03 (siE6 + siSet8)	81704490	80268672	12.3G	12G	0.03	97.91	94.31	51.7
SK04 (Gl2-Contro)	83973274	82477644	12.6G	12.4G	0.03	96.85	91.88	51.09
SK05 (siE6)	73744256	72293398	11.1G	10.8G	0.02	98.06	94.53	51.59
SK06(siE6 + siSet8)	75703744	74508840	11.4G	11.2G	0.03	97.91	94.27	51.59
SK07 (Gl2-Contro)	73376218	72123184	11G	10.8G	0.03	97.79	93.98	51.01
SK08(siE6)	67527940	66287472	10.1G	9.9G	0.03	97.79	94	51.77
SK09(siE6 + siSet8)	70788308	69651182	10.6G	10.4G	0.02	97.96	94.41	51.85

(S2) Mapped reads

Sample name	SK01	SK02	SK03	SK04	SK05	SK06	SK07	SK08	SK09
**Total reads**	70026436	72178864	80268672	82477644	72293398	74508840	72123184	66287472	69651182
**Total mapped reads**	67199286	68820950	76410802	77938064	68974080	71222798	69049858	63108728	66713024
**Uniquely mapped reads**	65875466	67520864	74949666	76379214	67677624	69868010	67692256	61917270	65408354
**Multiple mapped reads**	1323820	1300086	1461136	1558850	1296456	1354788	1357602	1191458	1304670
**Total mapping rate**	95.96%	95.35%	95.19%	94.50%	95.41%	95.59%	95.74%	95.21%	95.78%
**Uniquely mapping rate**	94.07%	93.55%	93.37%	92.61%	93.62%	93.77%	93.86%	93.41%	93.91%
**Multiple mapping rate**	1.89%	1.80%	1.82%	1.89%	1.79%	1.82%	1.88%	1.80%	1.87%

Supplementary - 4 : Original Western blot

Fig 1C

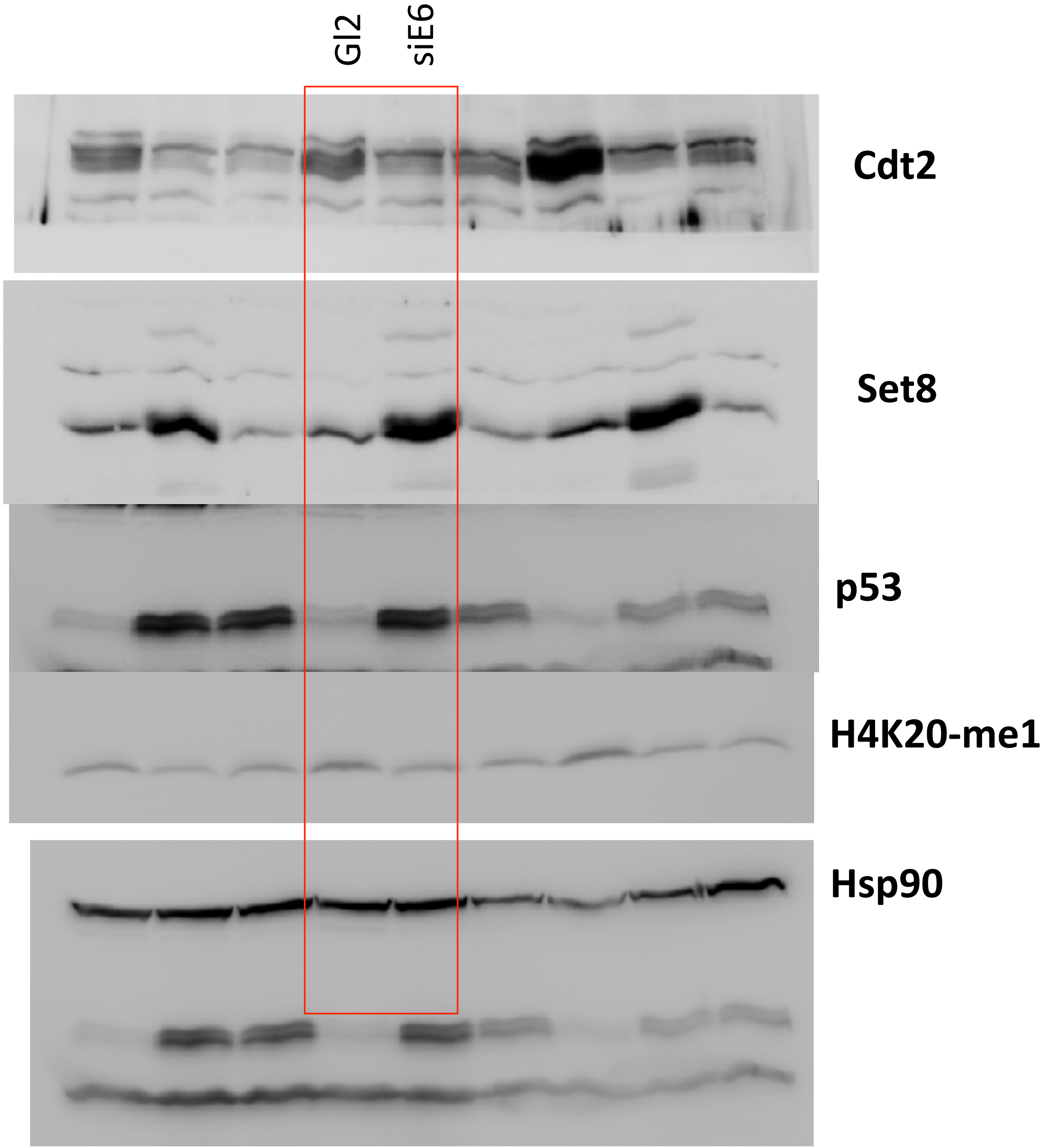

Quantifications
presented in the main figure

Fig 1D

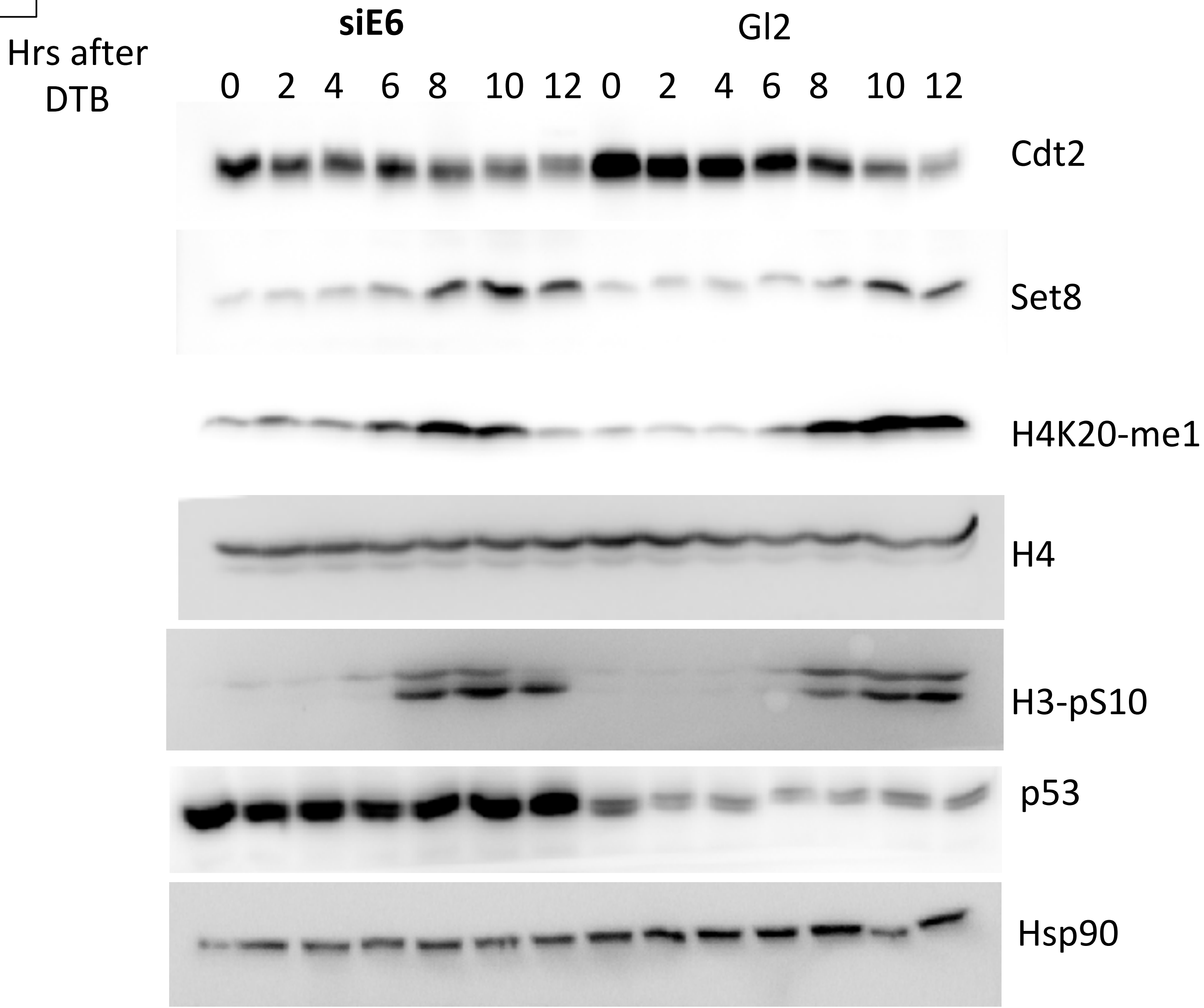

siE6
Gl2
Hrs after DTB
0 2 4 6 8 10 12 0 2 4 6 8 10 12
Cdt2
Set8
H4K20-me1
H4
H3-pS10
p53
Hsp90

Fig- 1D – Quantification data

	siE6								Gl2						
	**0**	**2**	**4**	**6**	**8**	**10**	**12**		**0**	**2**	**4**	**6**	**8**	**10**	**12**
**Cdt2**	1.37	0.77	0.72	0.78	0.61	0.57	0.48		**1.00**	0.96	1.19	1.28	0.98	0.79	0.37
**Set8**	1.44	1.66	1.82	2.94	5.32	6.84	4.72		**1.00**	0.98	1.48	1.71	2.50	7.59	4.60
**H4K20-me1**	2.49	3.37	2.88	5.33	8.12	6.03	1.64		**1.00**	0.95	1.20	4.38	10.49	15.59	10.58
**H4**	1.00	1.00	1.00	1.00	1.00	1.00	1.00		**1.00**	1.00	1.00	1.00	1.00	1.00	1.00
**H3-pS-10**	0.52	1.24	0.98	3.08	18.84	20.23	11.63		**1.00**	0.48	0.85	4.01	14.97	23.69	19.10
**p53**	3.87	2.31	2.27	2.05	2.00	2.11	1.85		**1.00**	0.55	0.74	0.54	0.57	1.05	0.73
**Hsp90**	0.62	0.97	0.81	0.94	0.94	0.87	0.85		**1.00**	1.18	1.49	1.77	2.02	1.43	1.49

Table-1: Values indicating densitometry analysis for each probed band at different time points corresponding to Fig.-1D. Each timepoint was normalised first to respective H4 measurmets and then to the measurement at 0 time point of Gl2

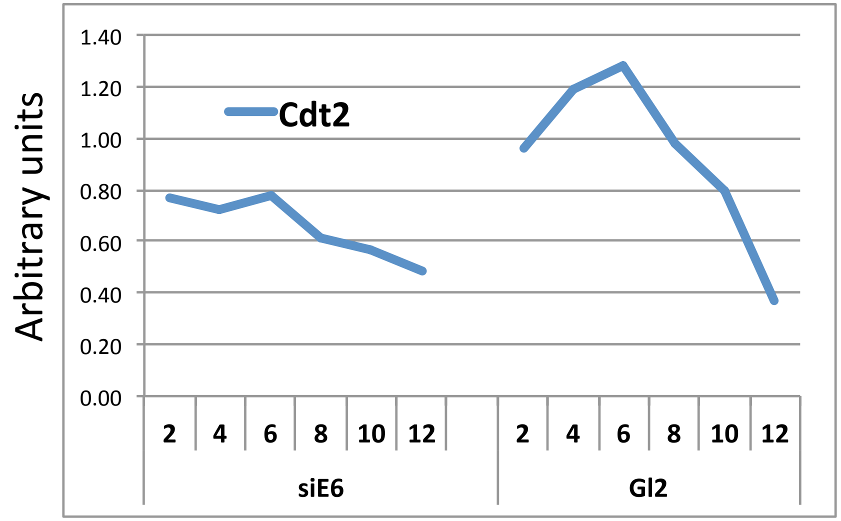

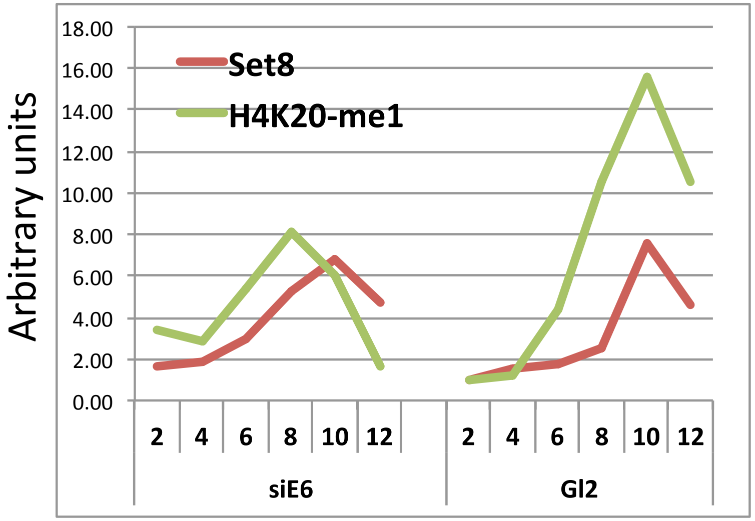

- Plots indicating the levels of Cdt2, Set8 and H4K20-me1 accumulation at different time points corresponding to Fig-1D . 0 -time point was eliminated because of aberrant values.

Fig 1E

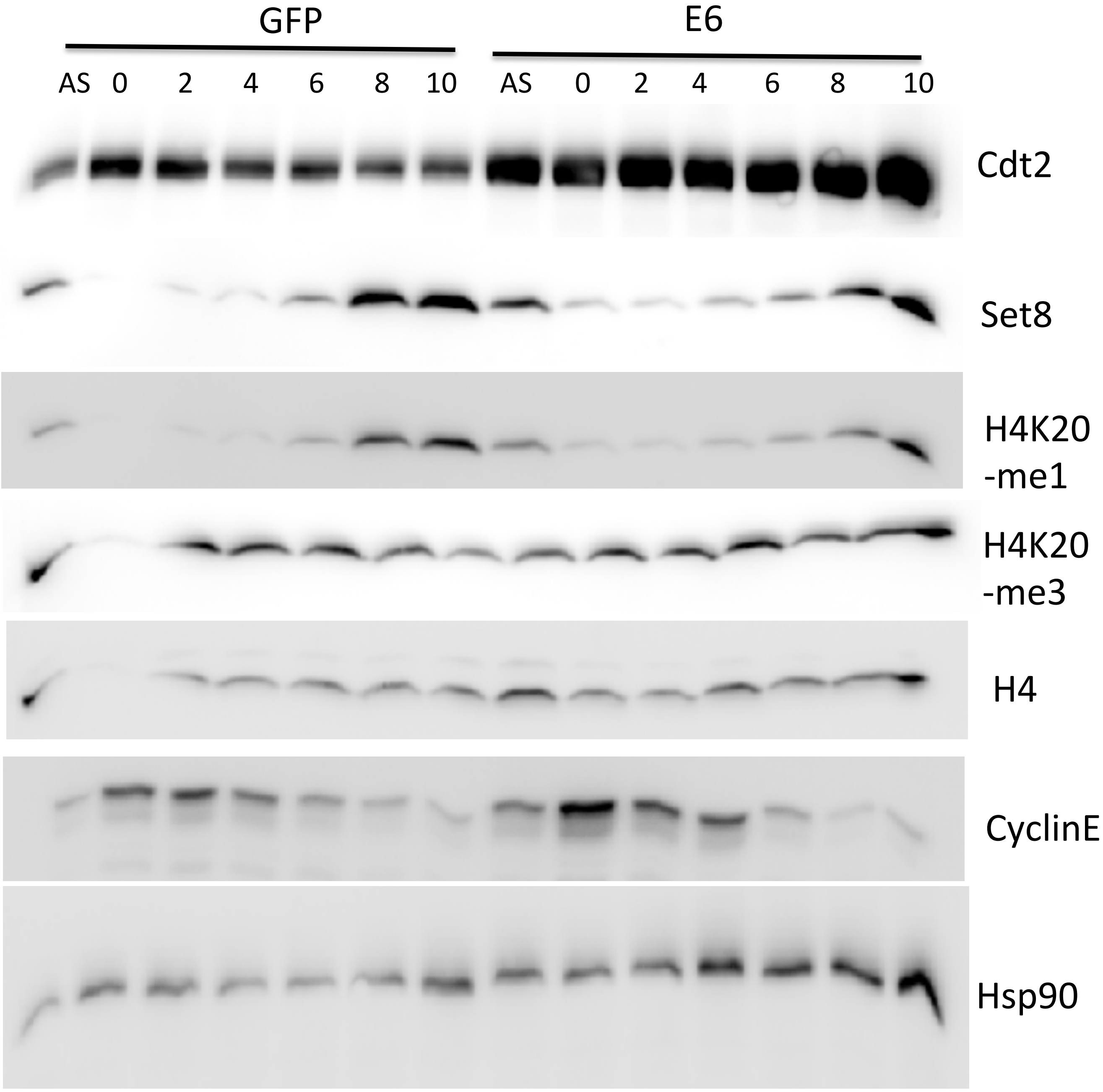
GFP
E6
AS 0 2 4 6 8 10
AS 0 2 4 6 8 10
Cdt2
Set8
H4K20
-me1
H4K20
-me3
H4
CyclinE
Hsp90

Fig- 1E – Quantification data

	GFP							E6					
	**AS**	**2**	**4**	**6**	**8**	**10**		**AS**	**2**	**4**	**6**	**8**	**10**
**Cdt2**	1.00	2.79	1.59	1.42	1.16	1.29		1.24	3.03	1.63	2.03	1.87	2.07
**Set8**	1.00	0.20	0.28	0.87	2.69	2.94		0.91	0.21	0.29	0.55	1.10	2.05
**H4K20-me[illegible]**	1.00	0.27	0.31	0.91	3.16	3.85		0.95	0.26	0.35	0.60	1.27	2.57
**H4K20-me[illegible]**	1.00	1.67	1.43	1.11	0.79	0.49		0.40	1.16	0.90	0.71	0.72	0.74
**H4**	1.00	1.00	1.00	1.00	1.00	1.00		1.00	1.00	1.00	1.00	1.00	1.00
**Cyclin E**	1.00	8.18	4.17	2.34	1.32	0.75		2.03	5.97	2.23	1.25	0.36	0.37
**Hsp90**	1.00	3.64	1.61	1.15	1.34	2.82		1.17	2.25	2.00	2.27	2.29	3.80

Table-2: Values indicating densitometry analysis for each probed band at different time points corresponding to Fig.-1E. Each timepoint was normalised first to respective H4 measurmets and then to the measurement at Asynchronous (AS) time point of GFP transfected cells.

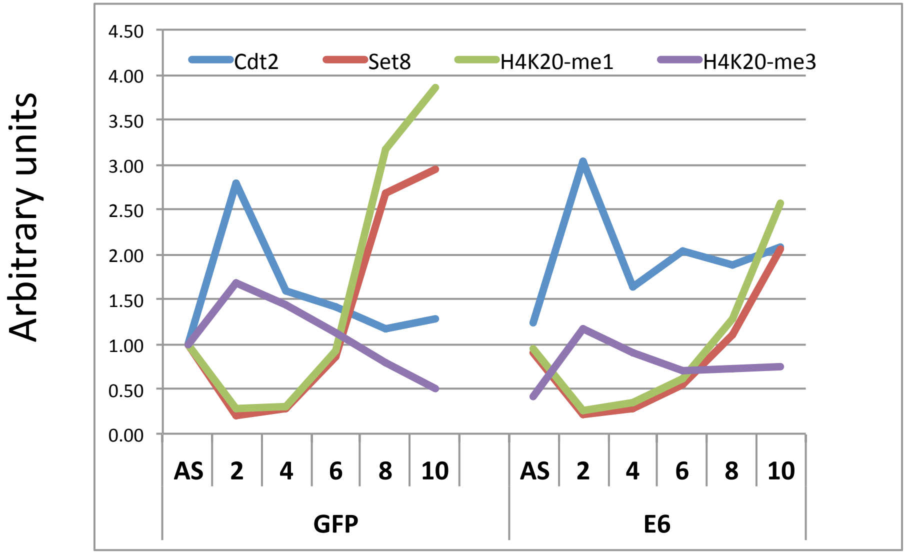

Plots indicating the levels of Cdt2, Set8,H4K20-me1 and H4K20-me3 accumulation at different time points corresponding to Fig-1D . 0 -time point was eliminated because of aberrant values

Fig 2A

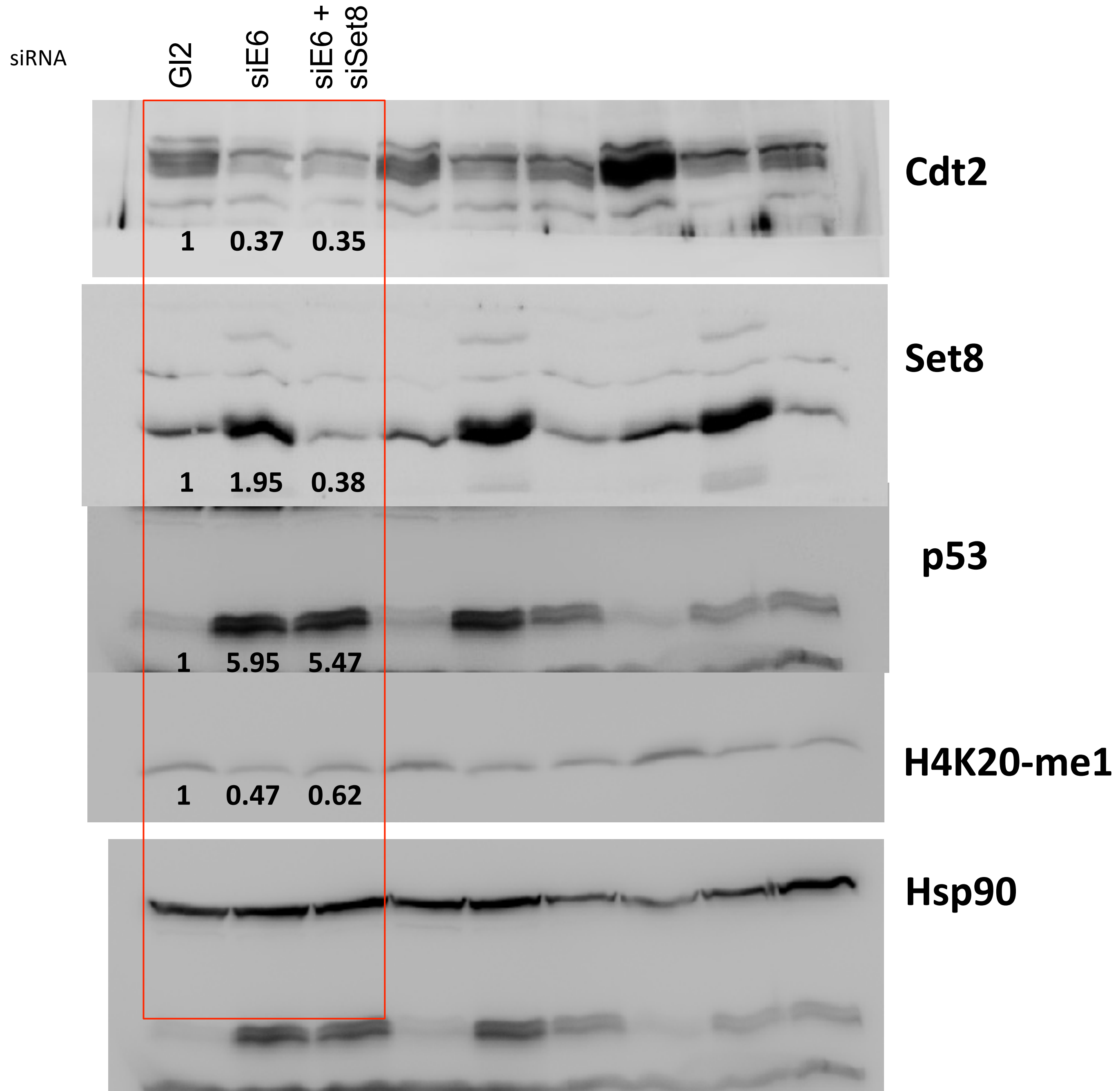
siRNA
Gl2
siE6
siE6 + siSet8
Cdt2
1 0.37 0.35
Set8
1 1.95 0.38
p53
1 5.95 5.47
H4K20-me1
1 0.47 0.62
Hsp90

Fig- 4B

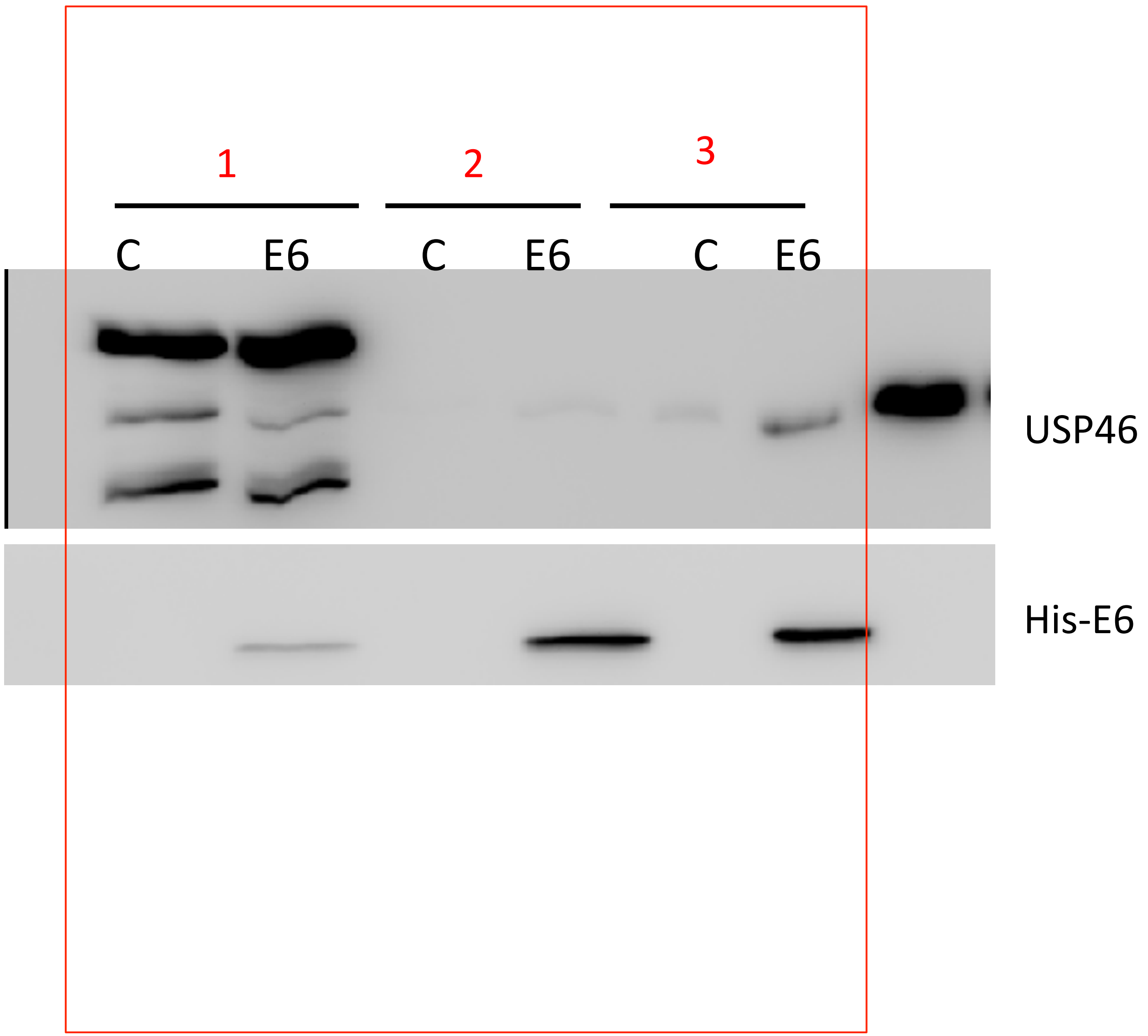

Immunoprecipitation experiment

FIG-5B

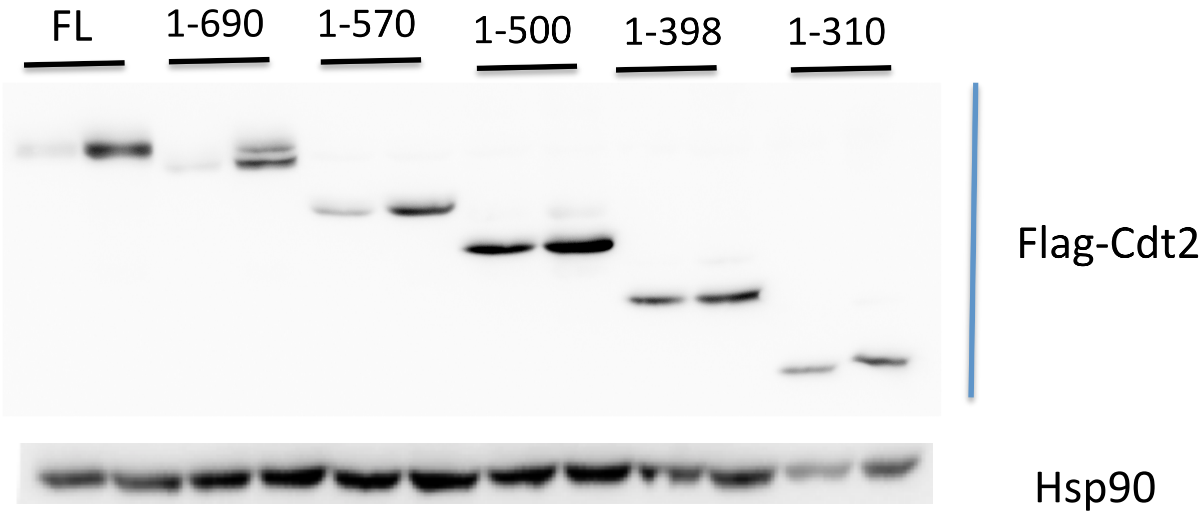

FIG-5B_inset

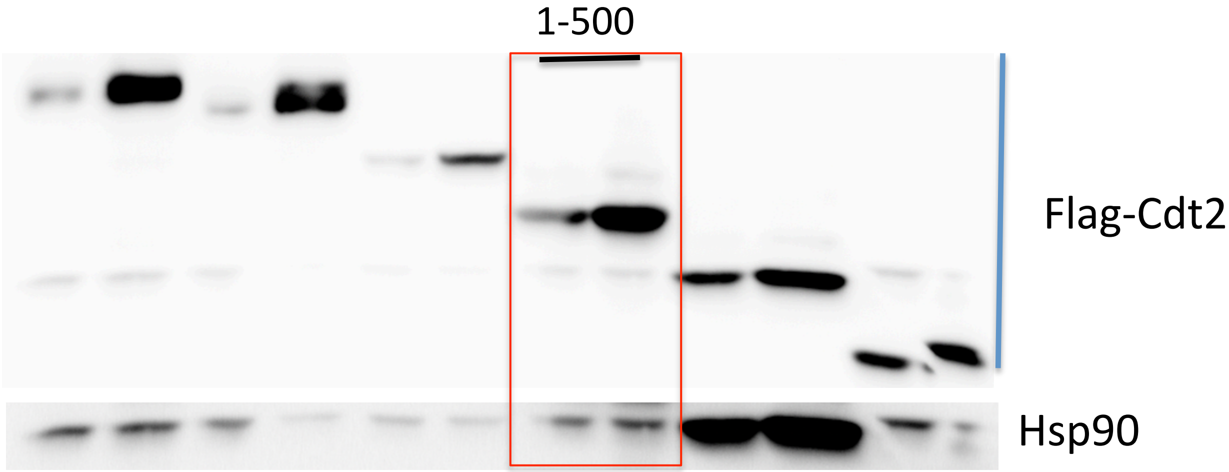

Fig- 5B – Quantification data

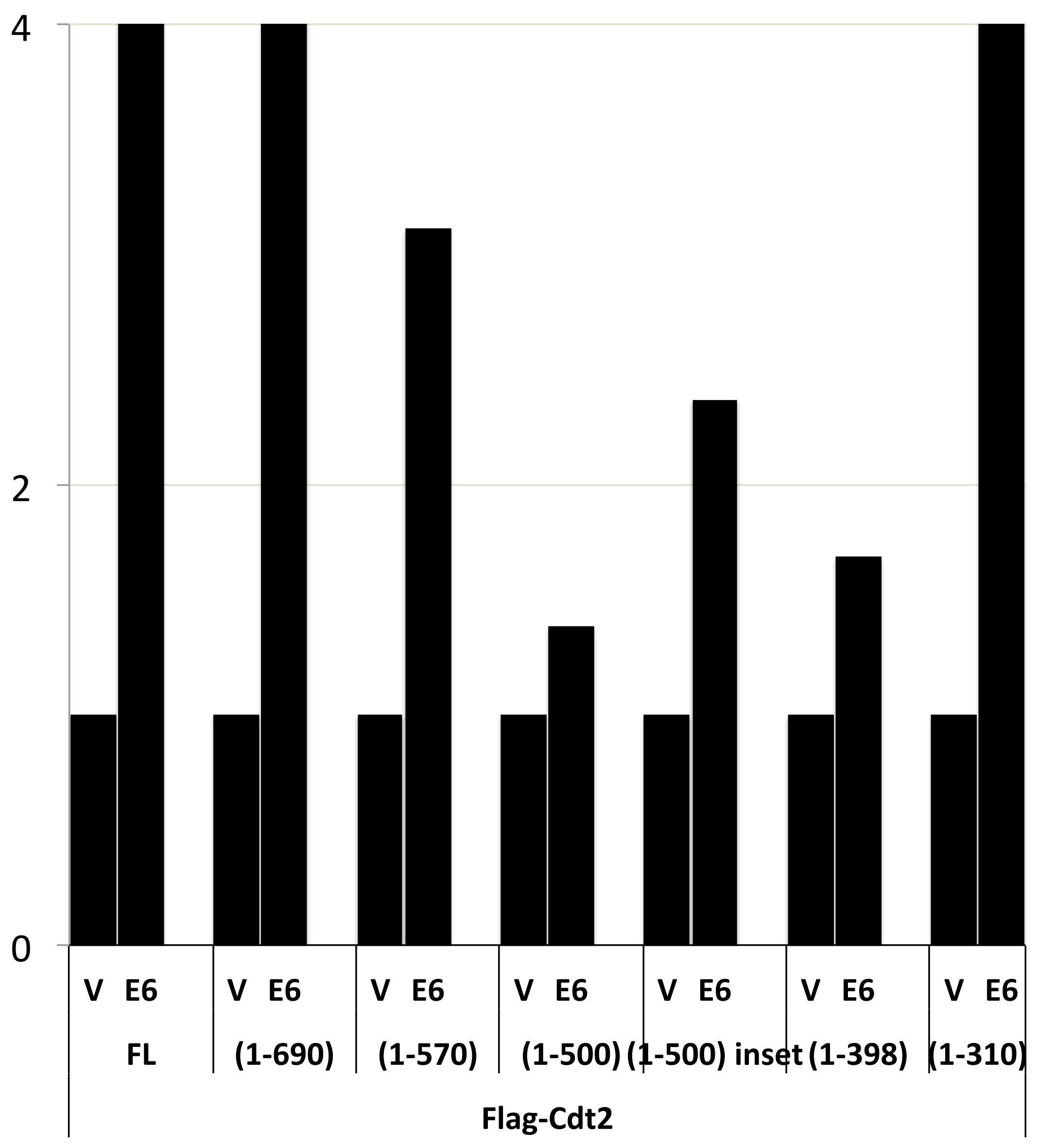

FIG-5D

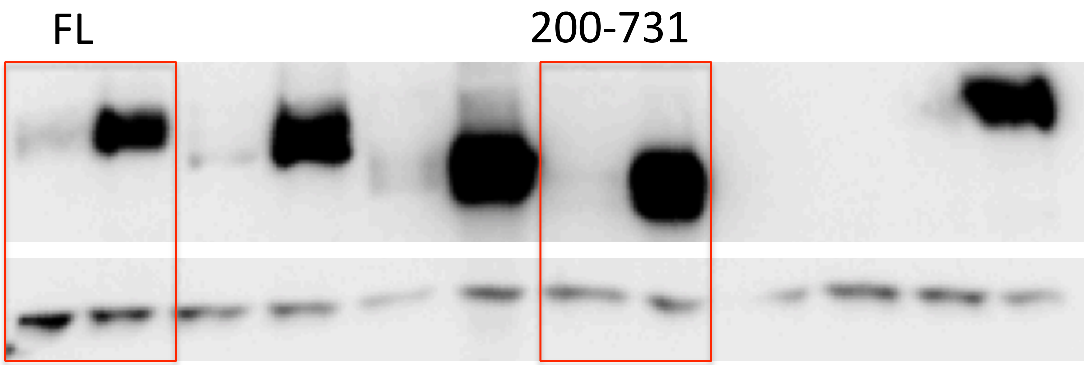

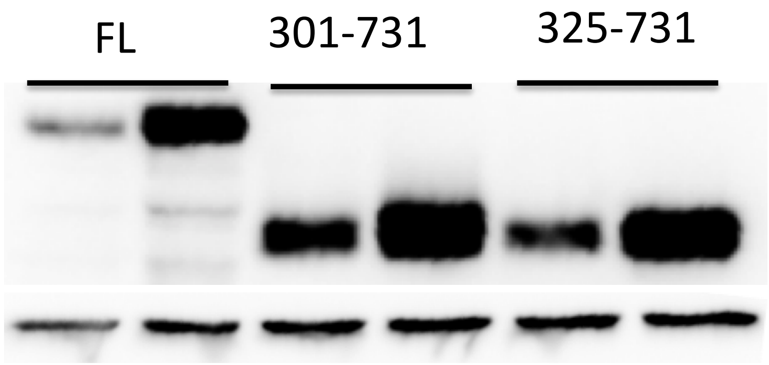

Fig- 5D – Quantification data

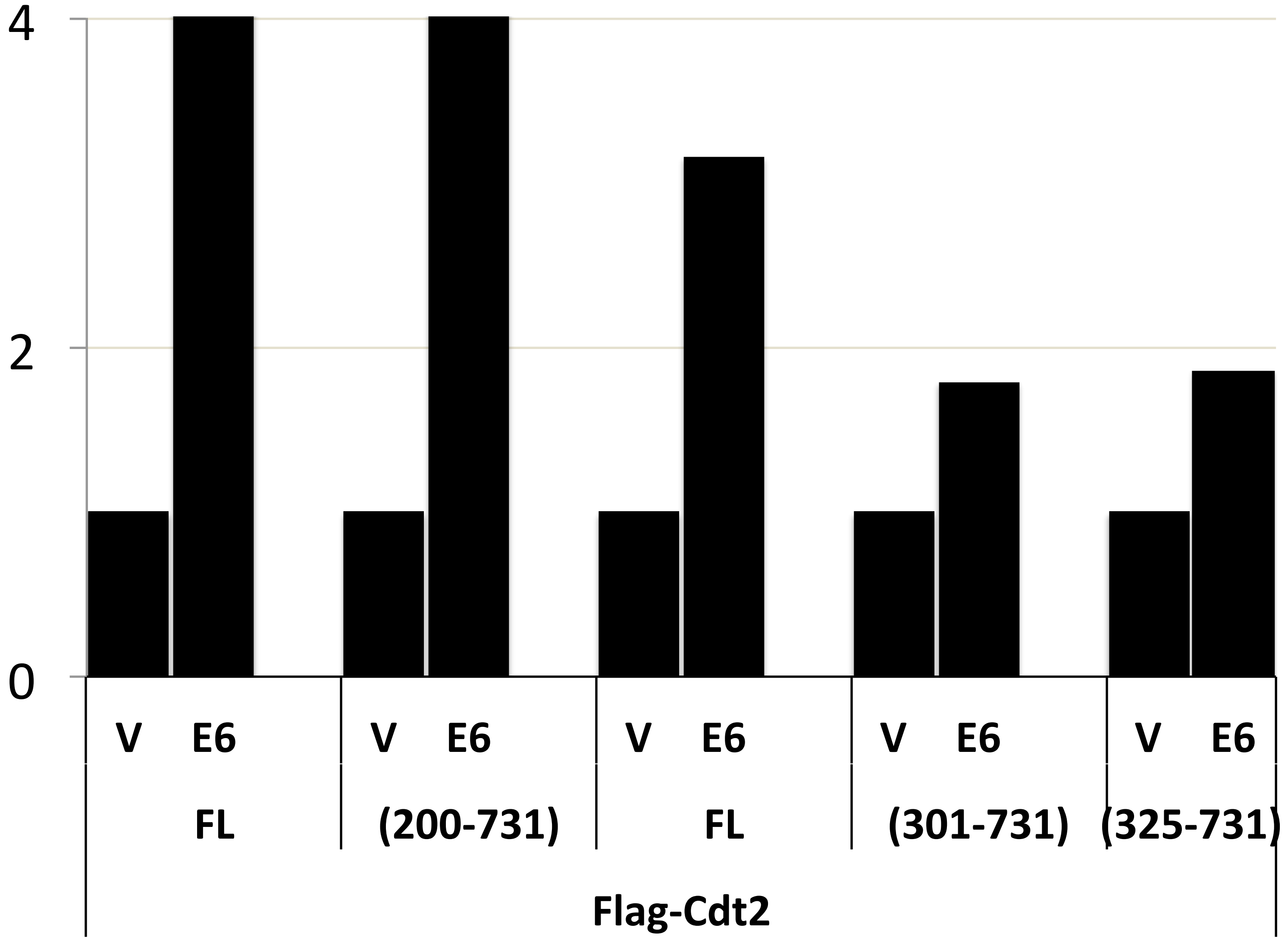

FIG-5F

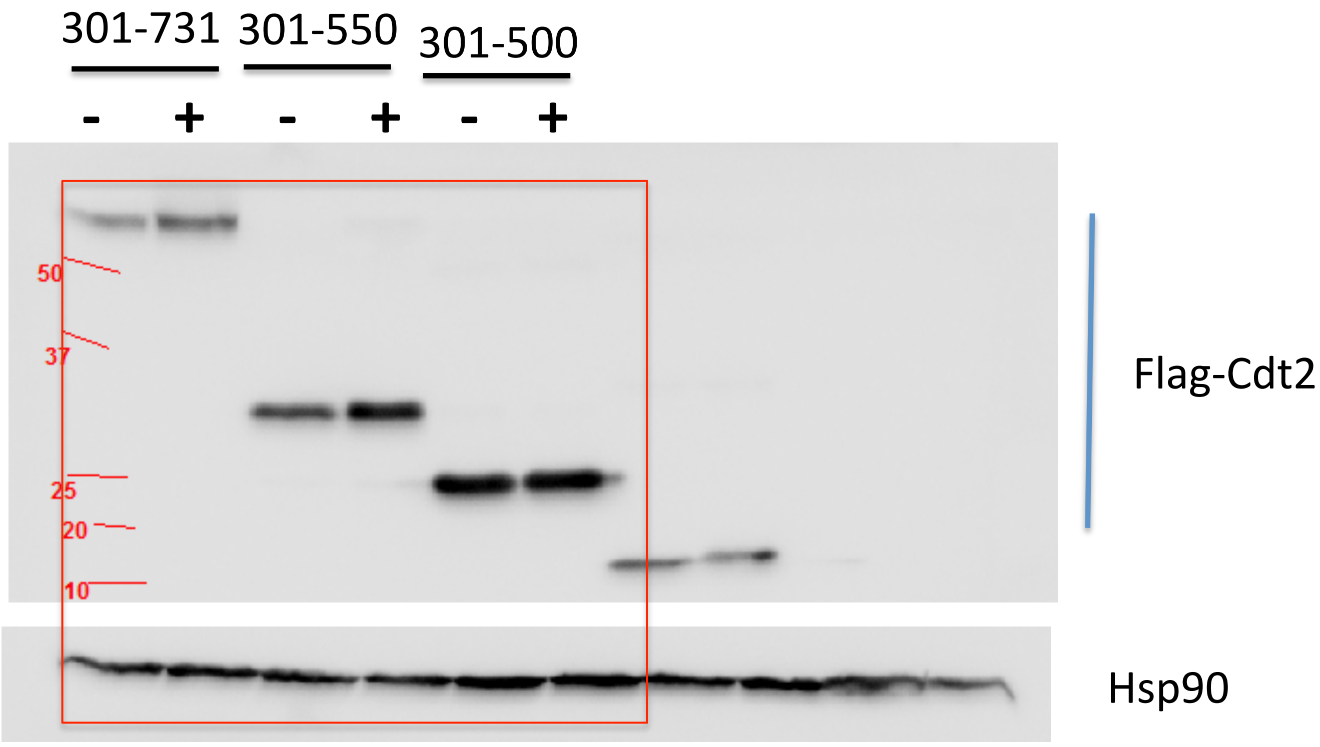

Quantifications
presented in the main figure

FIG-5H

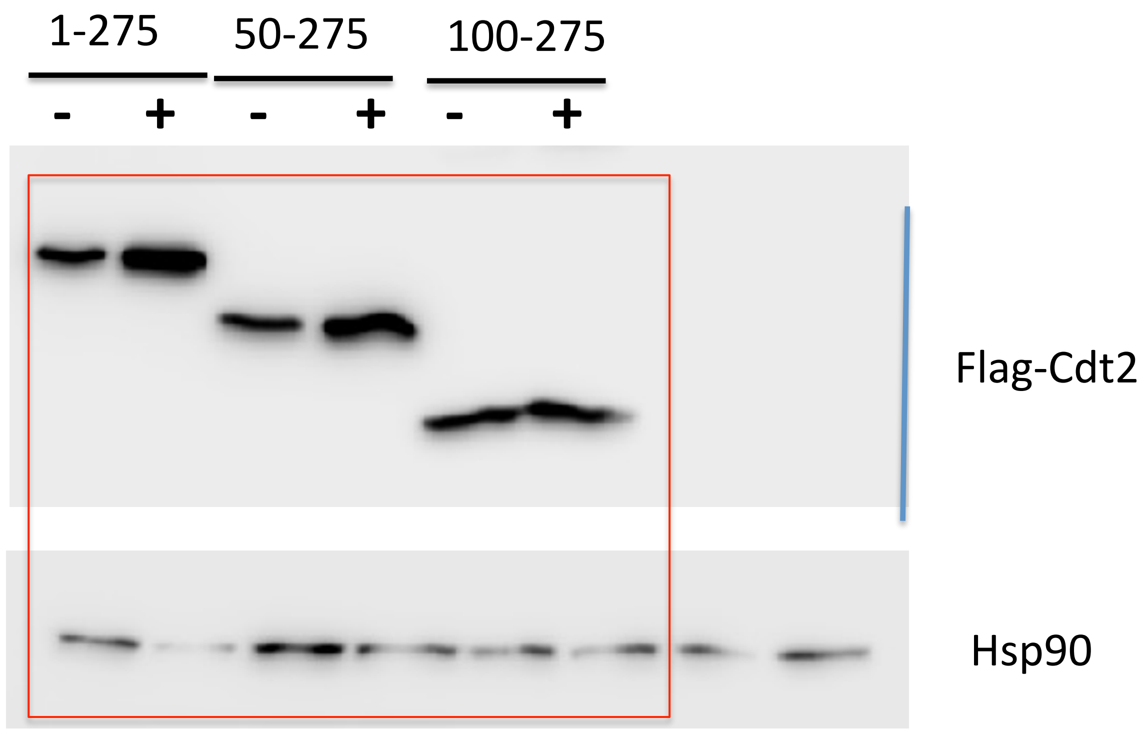

Quantifications
presented in the main figure
